# Genome-Wide Methylation Profiling of lncRNAs Reveals a Novel Progression-Related and Prognostic Marker for Colorectal Cancer

**DOI:** 10.3389/fonc.2021.782077

**Published:** 2022-01-20

**Authors:** Shujuan Lin, Simeng Gu, Sangni Qian, Yaxin Liu, Jinghao Sheng, Qilong Li, Jinhua Yang, Xiaojiang Ying, Zhenjun Li, Mengling Tang, Jianbing Wang, Kun Chen, Mingjuan Jin

**Affiliations:** ^1^ Department of Epidemiology and Biostatistics at School of Public Health and the Second Affiliated Hospital, Zhejiang University School of Medicine, Hangzhou, China; ^2^ Department of Environmental Health, Institute of Endemic Diseases, Zhejiang Provincial Center for Disease Control and Prevention, Hangzhou, China; ^3^ Institute of Environmental Medicine, and Cancer Center of the First Affiliated Hospital, Zhejiang University School of Medicine, Hangzhou, China; ^4^ Department of Screening, Jiashan Institute of Cancer Prevention and Treatment, Jiashan, China; ^5^ Department of Anorectal Surgery, Shaoxing People’s Hospital, Shaoxing, China; ^6^ Department of Epidemiology and Biostatistics at School Public Health and the Fourth Affiliated Hospital, Zhejiang University School of Medicine, Hangzhou, China; ^7^ Department of Epidemiology and Biostatistics at School of Public Health and National Clinical Research Center for Child Health of the Children’s Hospital, Zhejiang University School of Medicine, Hangzhou, China

**Keywords:** colorectal cancer, colorectal adenoma, DNA methylation, long noncoding RNA, prognosis, progression, *DLX6-AS1*

## Abstract

Sporadic colorectal cancer (CRC) develops principally through the adenoma-carcinoma sequence. Previous studies revealed that DNA methylation alterations play a significant role in colorectal neoplastic transformation. On the other hand, long noncoding RNAs (lncRNAs) have been identified to be associated with some critical tumorigenic processes of CRC. Accumulating evidence indicates more intricate regulatory relationships between DNA methylation and lncRNAs in CRC. Nevertheless, the methylation alterations of lncRNAs at different stages of colorectal carcinogenesis based on a genome-wide scale remain elusive. Therefore, in this study, we first used an Illumina MethylationEPIC BeadChip (850K array) to identify the methylation status of lncRNAs in 12 pairs of colorectal cancerous and adjacent normal tissues from cohort I, followed by cross-validation with The Cancer Genome Atlas (TCGA) database and the Gene Expression Omnibus (GEO) database. Then, the abnormal hypermethylation of candidate genes in colorectal lesions was successfully confirmed by MassARRAY EpiTYPER in cohort II including 48 CRC patients, and cohort III including 286 CRC patients, 81 advanced adenoma (AA) patients and 81 nonadvanced adenoma (NAA) patients. *DLX6-AS1* hypermethylation was detected at all stages of colorectal neoplasms and occurred as early as the NAA stage during colorectal neoplastic progression. The methylation levels were significantly higher in the comparisons of CRC vs. NAA (*P* < 0.001) and AA vs. NAA (*P* = 0.004). Moreover, the hypermethylation of *DLX6-AS1* promoter was also found in cell-free DNA samples collected from CRC patients as compared to healthy controls (*P*_adj_ = 0.003). Multivariate Cox proportional hazards regression analysis revealed *DLX6-AS1* promoter hypermethylation was independently associated with poorer disease-specific survival (HR = 2.52, 95% CI: 1.35-4.69, *P *= 0.004) and overall survival (HR = 1.64, 95% CI: 1.02-2.64, *P *= 0.042) in CRC patients. Finally, a nomogram was constructed and verified by a calibration curve to predict the survival probability of individual CRC patients (C-index: 0.789). Our findings indicate *DLX6-AS1* hypermethylation might be an early event during colorectal carcinogenesis and has the potential to be a novel biomarker for CRC progression and prognosis.

## Introduction

Colorectal cancer (CRC) is the third most commonly diagnosed cancer and the second leading cause of cancer-related death worldwide, with an estimated 1.9 million new cases and 935,000 deaths in 2020 (1). The majority of CRC cases are sporadic and develop principally through the adenoma-carcinoma sequence (2). It is well established that the gradual accumulation of multiple genetic and epigenetic changes plays a key role in the initiation and progression of colorectal carcinogenesis (3). In addition to conventional genetic variants, the regulatory contribution of epigenetic alterations has also been identified as a causative factor during cancer initiation and progression.

To date, aberrant DNA methylation, primarily in the form of hypermethylated or hypomethylated CpG dinucleotides within the genome, is one of the most extensively studied epigenetic alterations in human cancer (4). In particular, hypermethylation of gene promoter regions, which is frequently characterized by transcriptional silencing, remains the most dominant phenomenon during cancer development (5). Many studies have reported DNA methylation changes in cancer-related genes in CRC using genome-wide-based approaches or candidate gene strategies (6–8). Notably, these aberrant methylation alterations occur more frequently at the early stages of neoplastic progression (6). Indeed, hierarchical hypermethylation patterns of CRC-related suppressor genes, such as *SFRP2*, *SEPT9* and *MPPED2*, have been observed throughout the progression stages of colorectal carcinogenesis (9–11). Taken together, these findings indicate that abnormal changes in DNA methylation might be hallmarks of CRC initiation and progression. DNA hypermethylation might be one of the first detectable neoplastic alterations associated with carcinogenesis.

Long noncoding RNAs (lncRNAs) are defined as transcripts > 200 nucleotides in length without protein-coding capacity (12). Currently, these former so-called useless transcripts have been proven to be important regulators involved in biological, developmental, and pathological processes (13, 14). Remarkably, accumulating evidence supports more intricate regulatory relationships between DNA methylation and lncRNAs (15, 16). For instance, by performing an integrated analysis of epigenome and transcriptome data, Miller-Delaney et al. revealed that differential methylation might play an important role in the transcriptional regulation of lncRNAs in human temporal lobe epilepsy (17). He et al. identified 18 lncRNAs involved in methylation modifications that contributed to the tumorigenesis and development in glioma (16). Nevertheless, methylation studies of lncRNAs in CRC have largely been based on candidate gene strategy (18, 19). LncRNA methylation as biomarkers of CRC identified based on a genome-wide scale remain elusive.

Therefore, in this study, we first used an Illumina MethylationEPIC BeadChip (850K array) to identify the methylation status of lncRNAs in CRC. Then, we performed a technical validation of six candidate genes with MassARRAY EpiTYPER in CRC, followed by a comprehensive study to analyze the *DLX6-AS1* methylation pattern at different stages of colorectal neoplasms, from nonadvanced adenoma (NAA) to advanced adenoma (AA) to colorectal carcinoma. Furthermore, we evaluated the *DLX6-AS1* methylation levels in peripheral blood leucocyte DNA and analyzed their consistency with local lesions from the same patient. The methylation status of the *DLX6-AS1* promoter in cell-free DNA (cfDNA) of CRC patients was also evaluated. In addition, we performed survival analysis to clarify the prognostic role of methylated *DLX6-AS1* in CRC prognosis. A nomogram was established to predict the survival rate for CRC patients.

## Materials and Methods

### Study Design and Participants

**Figure 1:**
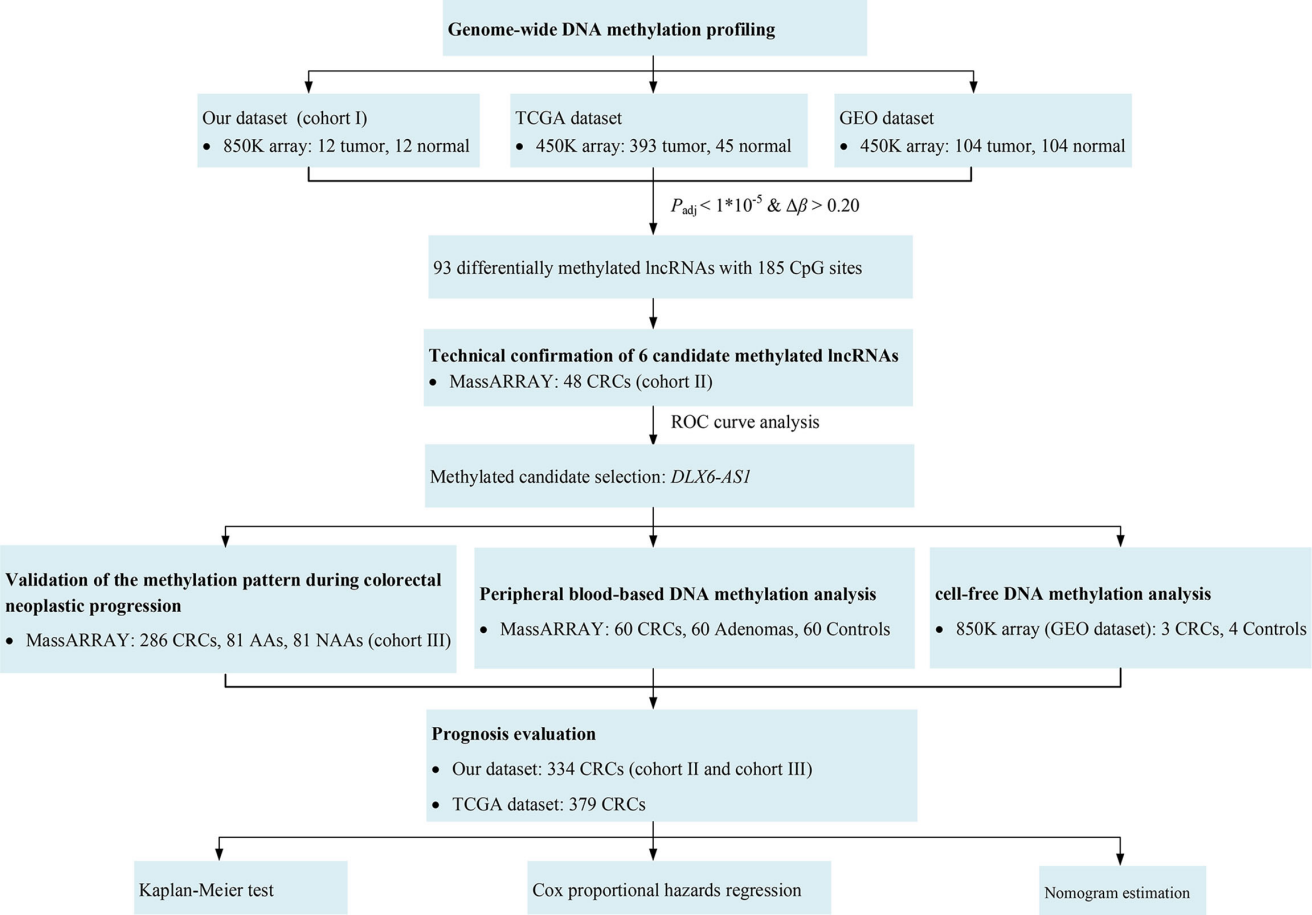
Study flowchart. AA, advanced adenoma; CRC, colorectal cancer; NAA, nonadvanced adenoma.

A flowchart for this study is shown in **Figure 1**. Briefly, this study was carried out in three cohorts. First, a genome-wide methylation scan by 850K array on cancerous and paired normal tissues from 12 CRC patients in cohort I was performed, followed by cross-validation using DNA methylation data from the TCGA database (https://cancergenome.nih.gov) and the GEO database (https://www.ncbi.nlm.nih.gov/geo/). The DNA methylation data from the TCGA and the GEO were generated using an Illumina HumanMethylation450 BeadChip (450K array) in 438 CRC tissue samples (393 tumor, 45 normal) and 208 CRC tissue samples (104 tumor, 104 normal), respectively. An overview of the external datasets used in this study is shown in **Supplementary Table S1**. Then, 48 pairs of CRC tissue samples from cohort II were tested. Additionally, the methylation levels of *DLX6-AS1* were further validated in cohort III, which consisted of 286 CRC patients, 81 AA patients and 81 NAA patients. The characteristics of the participants in each cohort subjected to the tissue-based methylation analysis are shown in **Table 1**.

**Table 1:** Basic characteristics of the study participants.

Characteristic	Cohort I	Cohort II	Cohort III		
	CRC (n = 12)	CRC (n = 48)	CRC (n = 286)	AA (n = 81)	NAA (n = 81)
Age, mean ± SD	63.50 ± 4.15	62.81 ± 9.28	64.88 ± 11.82	62.79 ± 6.64	63.47 ± 6.70
Sex, n (%)					
Male	7 (58.33)	24 (50.00)	171 (59.79)	41 (50.62)	40 (49.38)
Female	5 (41.67)	24 (50.00)	115 (40.21)	40 (49.38)	41 (50.62)
Location, n (%)					
Colon	6 (50.00)	22 (45.83)	133 (46.50)	59 (72.84)	60 (74.07)
Rectum	6 (50.00)	26 (54.17)	153 (53.50)	22 (27.16)	21 (25.93)
Stage, n (%)					
I	3 (25.00)	12 (25.00)	48 (16.78)		
II	3 (25.00)	12 (25.00)	95 (33.22)		
III	3 (25.00)	12 (25.00)	114 (39.86)		
IV	3 (25.00)	12 (25.00)	29 (10.14)		

AA, advanced adenoma; CRC, colorectal cancer; NAA, nonadvanced adenoma; SD, standard deviation.

To evaluate the DNA methylation levels in peripheral blood, we randomly sampled 60 CRC patients and 60 adenoma patients with complete tissue-based DNA methylation data from cohort II and cohort III, and 60 healthy controls from a population-based cohort. The DNA methylation status of the same region as measured in tissue samples was tested in each sample of peripheral blood leucocyte DNA. The characteristics of the participants subjected to the peripheral blood-based methylation analysis are shown in **Supplementary Table S2**.

To evaluate the DNA methylation levels in cfDNA, the DNA methylation data generated by 850K array in 7 cfDNA samples (3 CRCs, 4 healthy controls) were obtained from GEO database (**Supplementary Table S1**).

To evaluate the influence of *DLX6-AS1* methylation on survival, CRC patients with successfully measured DNA methylation data in our cohort II and cohort III were pooled together, and CRC patients with both available methylation data and survival information from the TCGA database were used as an external validation.

CRC patients from Shaoxing People’s Hospital were enrolled between January 2015 and July 2018. Participants with AA or NAA and healthy controls were selected from an ongoing population-based cohort since 1989 in Jiashan County, which has been described previously (11). All participants were ethnic Han Chinese from Zhejiang Province and were pathologically confirmed, with no familial adenomatous polyposis (FAP), no previous history of CRC and no preoperative anticancer treatment. For each participant, histologically confirmed tissue samples, including a colorectal lesion (carcinoma or adenoma) and an adjacent normal mucosa sample, and peripheral blood samples were obtained. The adjacent normal mucosa was collected from the colonic mucosa 5 cm distal from the main neoplasm. Adenomas were classified as AA (any adenoma ≥ 1 cm, high-grade dysplasia, or with tubulovillous or villous histology) and NAA (adenomas < 1 cm without advanced histology) according to current guidelines (20). The TNM staging classification for CRC was determined according to the 7th edition of the American Joint Committee on Cancer (AJCC) cancer staging manual (21).

The study protocol was approved by the Medical Ethics Committee of Zhejiang University School of Medicine. Before basic information and sample collection, written informed consent was obtained from all recruited participants.

### DNA Extraction and Bisulfite Modification

Genomic DNA from fresh-frozen samples and peripheral blood leukocytes was isolated using a DNA Tissue Kit (TianLong Biotech, Xi’an, China) and a RelaxGene Blood DNA System (TianGen Biotech, Beijing, China), respectively. Bisulfite treatment was conducted on genomic DNA (500 ng) using the EZ Methylation Gold Kit (Zymo Research, Irvine, CA, USA). All procedures were conducted in accordance with the manufacturer’s instructions.

### Illumina Methylation Assay

Genome-wide DNA methylation profiling was analyzed using the 850K array in 12 pairs of cancerous and adjacent normal tissues according to the manufacturer’s instructions as described in a previous study (11). In this study, the raw array data were processed using the ChAMP package in R software for deriving the methylation level, which was generated as beta values (fraction methylation values between 0 and 1). We focused mainly on probes located in the promoter region of lncRNAs, which was defined as 1500 bp upstream and downstream from the transcription start site (TSS). The lncRNA annotation file was obtained from LNCipedia (https://hg19.lncipedia.org/) and the mapping procedure was conducted using the bedtools (22). Probes were selected on the basis of showing a difference in methylation of ≥ 0.20 and an adjusted *P* value (Benjamini-Hochberg method) < 0.05. To cross-validate the results based on our samples, the eligible methylation data in TCGA and GEO were obtained and analyzed. The detailed procedures of data processing have been supplemented in the **Supplementary Methods**. Due to the larger coverage of the 850K array as compared to 450K array, the new probes in 850K array were cross-validated by the average beta value of the promoter regions of the target genes in 450K array.

### Sequenom MassARRAY EpiTYPER Assay

The methylation levels of particular CpG sites located in the promoter region of candidate genes were verified using MassARRAY EpiTYPER (Sequenom, San Diego, CA). The schematic representation of each candidate gene is provided in the UCSC browser (http://genome.ucsc.edu). The primers were designed using EpiDesigner (http://epidesigner.com, **Supplementary Table S3**). The analyzed sequences are shown in **Supplementary Figures S1** **–** **6**. In some cases, fragments resulting from the T-cleavage reaction may contain small groups of adjacent CpG sites and are therefore referred to as “CpG units”. CpG sites that were outside of the mass spectrometry analytical window (low or high mass) were filtered out. The mass spectra were collected on a MassARRAY Compact MALDI-TOF system (Sequenom, BioMiao Biological Technology, Beijing, China), and the methylation proportions of individual units on the spectra were generated by EpiTYPER software (Sequenom, San Diego, CA). Methylation levels ranging from 0 (completely nonmethylated) to 1 (fully methylated) are presented. For each gene, CpG unites with missing values in more than 20% of the samples were removed, as well as samples with missing values in more than 20% of CpG unites. The average methylation value of all CpG units was calculated as a representation of the region-specific gene methylation level.

### Statistical Analysis

Statistical analyses were performed in R software (version 3.6.2, R Foundation for Statistical Computing, Vienna, Austria). Continuous variables are presented as the mean and standard deviation (SD), and categorical variables are presented as the frequency.

A paired Student’s *t* test was used to assess the differences in DNA methylation levels between colorectal lesion tissues and paired normal tissues. Analysis of variance (ANOVA) followed by Bonferroni’s posttest was used to examine significant differences between different groups. Pearson correlation analyses were used to evaluate the consistency of *DLX6-AS1* methylation levels between peripheral blood and local lesions of the same patients with CRC or adenoma. The performance of the mean methylation level of candidate genes in distinguishing colorectal lesion tissues from their adjacent normal tissues was tested by receiver operating characteristic (ROC) curve analysis, and the area under the curve (AUC), sensitivity, and specificity were calculated. In the survival analysis, we adopted the best Youden index based on the time-dependent ROC curve as an optimal cutoff to dichotomize the study patients into high-risk and low-risk groups. Survival differences between groups were assessed using the Kaplan-Meier test and compared by the log-rank test. Hazard ratios (HRs) and 95% confidence intervals (95% CIs) were calculated by univariate and multivariate Cox proportional hazards regression analyses. The multivariate analysis was adjusted for age, sex and TNM stage. A nomogram was established to predict the 1-, 2-, 3- and 4-year survival for CRC patients. Harrell’s concordance index (C-index) was measured to quantify the discrimination ability of the nomogram, while the calibration curves were used to evaluate whether the predicted survival probabilities were consistent with those observed. All analyses were carried out in a two-sided manner, with a *P* value < 0.05 regarded as statistically significant.

## Results

### Discovery of Differentially Methylated lncRNAs From Genome-Wide Profiling

**Figure 2:**
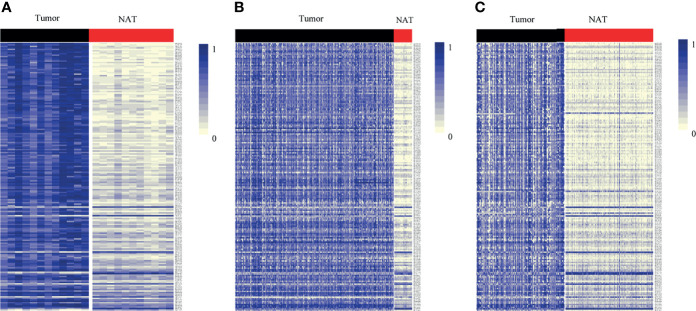
Heat map showing differentially methylated CpG sites mapping to the promoter of lncRNAs between lesion tissues and paired adjacent normal tissues from genome-wide profiling. Methylation profiling using **(A)** 850K array of our dataset. **(B)** 450K array from TCGA dataset. **(C)** 450K array from GEO dataset. Each column represents a sample. Each row represents the methylation level of an individual CpG sites, which is depicted as a color gradient ranging from light yellow (completely nonmethylated) to blue (fully methylated). NAT, histologically normal tissue adjacent to the lesion.

**Figure 3:**
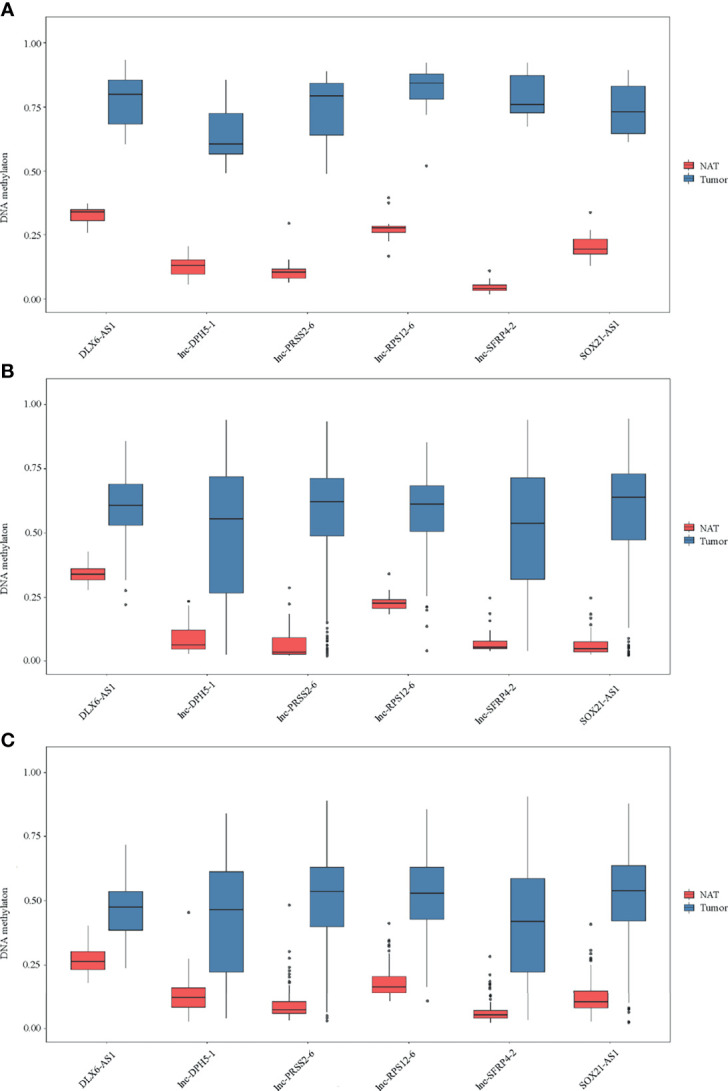
Methylation of six selected genes in cohort I. Methylation levels of *DLX6-AS1*, *lnc-DPH5-1*, *lnc-PRSS2-6*, *lnc-RPS12-6*, *lnc-SFRP4-2* and *SOX21-AS1* in CRC tissues and paired normal tissues are shown for **(A)** 850K array of our dataset. **(B)** 450K array of TCGA database. **(C)** 450K array of GEO database. NAT, histologically normal tissue adjacent to the lesion.

By DNA methylation profiling, a total of 185 differentially methylated CpG sites mapping to the promoter of lncRNAs (all with *P*_adj_ < 1*10^-5^ and *β* difference > 0.20) were identified by the 850K array generated from 12 pairs of colorectal cancerous and adjacent normal tissues, followed by cross-validation using DNA methylation data generated by the 450K array in CRCs from the TCGA database (tumor=393, normal=45) and GEO database (tumor=104, normal=104), respectively (**Supplementary Table S4**). Among them, 95.14% (176/185) of the identified CpG sites were significantly hypermethylated and 4.86% (9/185) were significantly hypomethylated. The methylation levels for each differentially methylated CpG sites are shown by heat maps (**Figure 2**). Among the list of CpG sites, we focused on six sites ranking on the top (cg24014202 in *DLX6-AS1*, cg18323466 in *lnc-DPH5-1*, cg08430489 in *lnc-PRSS2-6*, cg17722675 in *lnc-RPS12-6*, cg00159100 in *lnc-SFRP4-2*, cg27442308 in *SOX21-AS1*) for following technical confirmation analysis (**Figure 3**), which were considered as candidate biomarkers.

### Confirmation of Promoter Hypermethylation Status Using MassARRAY EpiTYPER

**Figure 4:**
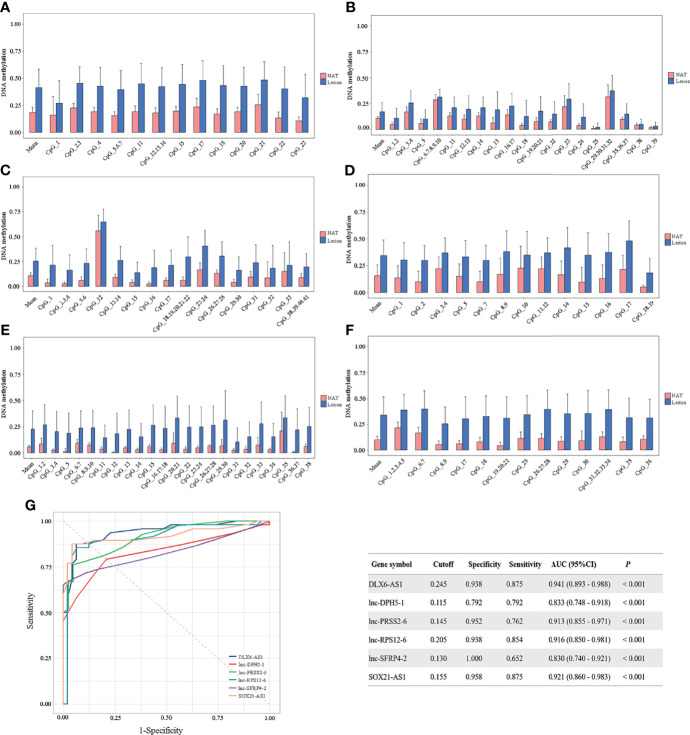
Methylation status of six selected candidates quantified by MassARRAY EpiTYPER among CRC patients in cohort II. Quantifications are shown for methylation levels of **(A)** *DLX6-AS1*, **(B)** *lnc-DPH5-1*, **(C)** *lnc-PRSS2-6*, **(D)** *lnc-RPS12-6*, **(E)** *lnc-SFRP4-2* and **(F)** *SOX21-AS1*. **(G)** The discriminative ability of the six selected genes between CRC tissues and adjacent normal tissues by ROC analysis. NAT, histologically normal tissue adjacent to the lesion.

To confirm the above findings, target regions covering the identified CpG units in the promoter of the above 6 candidate genes were amplified in 48 CRCs by MassARRAY EpiTYPER in cohort II. The methylation levels of the mean and individual CpG units for each gene, which were significantly higher in colorectal cancerous tissues than in adjacent normal tissues, are shown in bar plots (**Figures 4A–F**). Specifically, the increases in methylation status between cancerous and paired normal mucosa were found in 95.83% (46/48), 79.17% (38/48), 100% (42/42), 93.75% (45/48), 80.43% (37/46) and 91.67% (44/48) of CRCs, respectively, for *DLX6-AS1*, *lnc-DPH5-1*, *lnc-PRSS2-6*, *lnc-RPS12-6*, *lnc-SFRP4-2* and *SOX21-AS1* (**Supplementary Figure S7**).

ROC curve analyses revealed that methylation status of each individual genes could significantly distinguish primary carcinoma from adjacent normal mucosa, as measured by AUC value (*DLX6-AS1*: 0.941; *lnc-DPH5-1*: 0.833; *lnc-PRSS2-6*: 0.913; *lnc-RPS12-6*: 0.916; *lnc-SFRP4-2*: 0.830; *SOX21-AS1*: 0.921) (**Figure 4G**). Among them, *DLX6-AS1* showed a high discriminative performance and was therefore chosen for further validation.

### Elucidation of the Aberrant *DLX6-AS1* Methylation Pattern During Colorectal Neoplastic Progression

**Figure 5:**
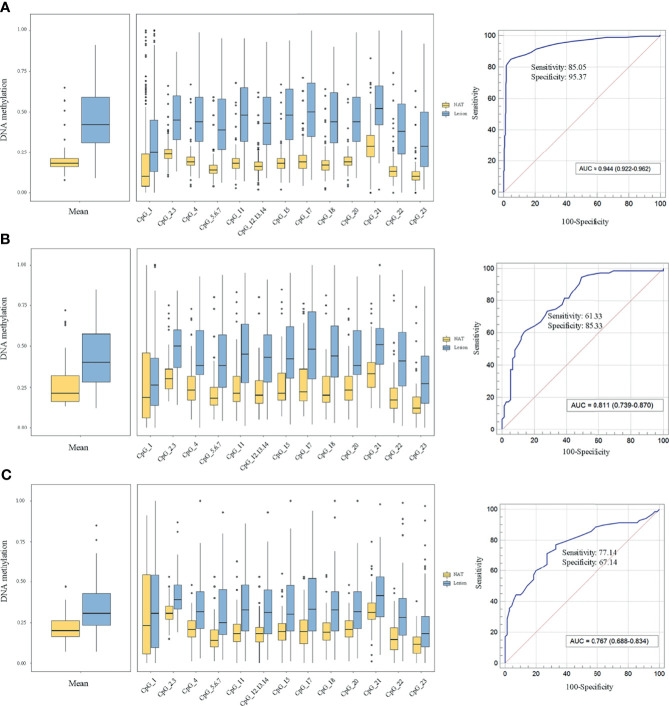
Methylation levels of *DLX6-AS1* quantified by MassARRAY EpiTYPER in cohort III and its discriminative ability. **(A)** Colorectal cancer (n = 281). **(B)** Advanced adenoma (n = 75). **(C)** Nonadvanced adenoma (n = 70). NAT, histologically normal tissue adjacent to the lesion.

To elucidate the *DLX6-AS1* methylation pattern during colorectal neoplastic progression, the methylation status was assessed in colorectal lesion tissues and adjacent normal tissues from 286 CRCs, 81 AAs and 81 NAAs in cohort III with MassARRAY EpiTYPER. Among them, 433 histologically confirmed colorectal lesion tissues (283 CRCs, 76 AAs and 74 NAAs) and 441 adjacent normal tissues (284 CRCs, 80 AAs and 77 NAAs) were successfully measured. *DLX6-AS1* hypermethylation was detected at all stages of colorectal neoplasms, even as early as the NAA stage. Compared to their adjacent normal tissues, 94.31% (265/281) of CRCs, 85.33% (64/75) of AAs and 80.00% (56/70) of NAAs presented higher *DLX6-AS1* methylation levels, with statistically significant differences (all *P* < 0.001) (**Figure 5** and **Supplementary Figure S8**). The mean *DLX6-AS1* methylation levels could distinguish primary lesions from their adjacent normal mucosa, with AUC values of 0.944 (95% CI: 0.922-0.962), 0.811 (95% CI: 0.739-0.870) and 0.767 (95% CI: 0.688-0.834) for CRC, AA and NAA, respectively (**Figure 5**). When comparing the *DLX6-AS1* methylation levels between different stages of colorectal lesions (**Table 2**), the *DLX6-AS1* promoter was revealed to be significantly hypermethylated between CRC vs. NAA (*P* < 0.001) and AA vs. NAA (*P* = 0.004) but not between CRC vs. AA (*P* = 1.000).

**Table 2:** *DLX6-AS1* methylation differences between colorectal cancer, advanced adenoma and nonadvanced adenoma patients.

CpG unit	CRC/AA/NAA, n	Methylation level			*P^a^*	CRC vs. AA		CRC vs. NAA		AA vs. NAA	
		CRC	AA	NAA		Δß	*P^b^*	Δß	*P^b^*	Δß	*P^b^*
Mean	283/76/74	0.45	0.44	0.35	**<0.001**	0.01	1.000	0.10	**<0.001**	0.09	**0.004**
CpG_1	270/69/70	0.32	0.32	0.33	0.951	-0.01	–	-0.01	–	0.00	–
CpG_2.3	283/76/74	0.47	0.49	0.42	**0.014**	-0.02	0.854	0.05	**0.045**	0.07	**0.016**
CpG_4	283/76/74	0.46	0.45	0.35	**<0.001**	**0.01**	1.000	0.11	**<0.001**	0.10	**0.002**
CpG_5.6.7	283/75/73	0.43	0.41	0.31	**<0.001**	**0.02**	1.000	0.11	**<0.001**	0.09	**0.013**
CpG_11	283/76/74	0.49	0.45	0.36	**<0.001**	**0.03**	0.669	0.13	**<0.001**	0.09	**0.015**
CpG_12.13.14	283/75/74	0.45	0.43	0.33	**<0.001**	**0.02**	1.000	0.11	**<0.001**	0.09	**0.010**
CpG_15	283/76/74	0.49	0.46	0.35	**<0.001**	**0.03**	0.659	0.14	**<0.001**	0.10	**0.003**
CpG_17	283/76/74	0.52	0.50	0.37	**<0.001**	**0.02**	1.000	0.15	**<0.001**	0.13	**0.001**
CpG_18	283/76/74	0.46	0.47	0.35	**<0.001**	**-0.01**	1.000	0.11	**<0.001**	0.12	**0.001**
CpG_20	283/76/74	0.46	0.45	0.35	**<0.001**	**0.01**	1.000	0.11	**<0.001**	0.10	**0.002**
CpG_21	281/74/74	0.54	0.51	0.42	**<0.001**	**0.03**	0.557	0.12	**<0.001**	0.09	**0.004**
CpG_22	283/76/74	0.40	0.43	0.33	**0.009**	-0.03	0.904	0.07	**0.028**	0.10	**0.012**
CpG_23	283/76/74	0.34	0.31	0.24	**0.001**	0.04	0.593	0.10	**0.001**	0.07	0.152

Bold values: Statistically significant. AA, advanced adenoma; CRC, colorectal cancer; NAA, nonadvanced adenoma.

a ANOVA.

b Bonferroni t test.

### Evaluation of *DLX6-AS1* Methylation Levels in Peripheral Blood and Their Consistency With Local Colorectal Lesions

To evaluate the potential of *DLX6-AS1* methylation as a noninvasive biomarker for the diagnosis of colorectal neoplasms, *DLX6-AS1* methylation levels were measured in the peripheral leucocyte DNA of 60 CRC patients, 60 adenoma patients and 60 healthy controls. However, there were no significant differences in peripheral blood-based *DLX6-AS1* methylation levels in multiple comparisons between CRC patients, adenoma patients and healthy controls (**Supplementary Table S5**). Even though some CpG units, such as CpG_2.3, reached a statistically significant level (*P* = 0.017), the methylation levels did not differ much across the different groups. When evaluating the consistency between peripheral blood and local lesions from the same patients (**Supplementary Table S6**), the Pearson correlation analysis showed poor correlations between matched peripheral blood and local lesions (*P* = 0.362 for CRCs and 0.893 for adenomas, respectively, in average methylation levels).

### *DLX6-AS1* Methylation in Cell-Free DNA Samples From Colorectal Cancer

To identify the methylation status of the *DLX6-AS1* promoter in cfDNA of CRC patients, we analyzed the methylation data generated by the 850K array in cfDNA from 3 CRC patients and 4 healthy controls in GEO dataset. Among the available 35 CpG sites in the promoter of *DLX6-AS1*, 18 significantly hypermethylated CpG sites were identified (*ß* difference > 0.20 and *P*_adj_ < 0.05). The mean methylation values were 0.40 and 0.16 in CRC patients and healthy controls, respectively (*P*_adj_ = 0.003). Details of the results are presented in **Supplementary Figure S9**.

### Analysis of the Association of the *DLX6-AS1* Methylation Status With CRC Prognosis

**Figure 6:**
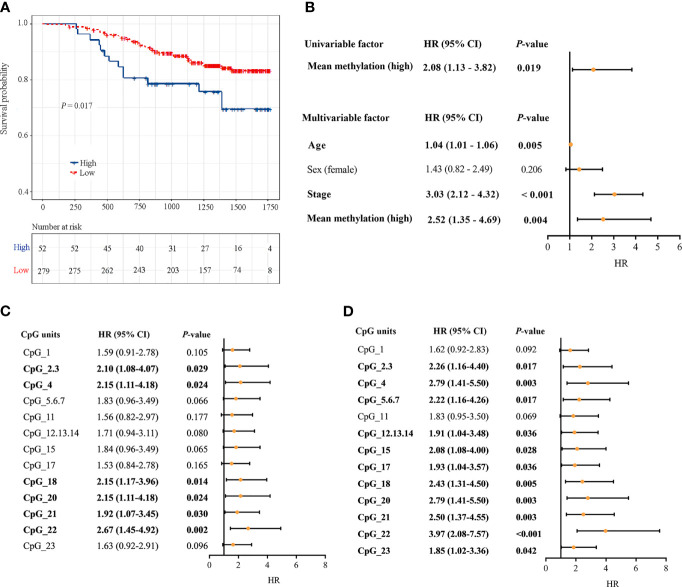
Associations between *DLX6-AS1* methylation levels and CRC-specific survival. **(A)** Kaplan-Meier estimation of the disease-specific survival of the entire set of CRC patients (N = 331) using the mean methylation levels of *DLX6-AS1*. The blue line indicates the group with a high methylation level. The red line indicates the group with a low methylation level. **(B)** Univariate and multivariate Cox regression analysis with the mean methylation level. Multivariate Cox regression analysis were adjusted for age, sex and TNM stage. **(C)** Univariate Cox regression analysis with the methylation status of individual CpG units within *DLX6-AS1* promoter. **(D)** Multivariate Cox regression analysis with the methylation status of individual CpG units within *DLX6-AS1* promoter. Orange solid dots represent the point estimation of the hazard ratio (HR) of disease-specific death, and the open-ended horizontal lines represent the 95% confidence intervals (CIs).

331 CRC patients with DNA methylation successfully measured by MassARRAY EpiTYPER were included. During follow-up, there were 53 CRC-specific deaths, and the median follow-up time was 3.60 years. Kaplan-Meier analysis revealed that patients with a high *DLX6-AS1* methylation status had poorer disease-specific survival (DSS) rates than those with a low methylation status (*P* = 0.017, **Figure 6A**). A multivariate Cox regression model considering the most relevant risk factors, including age, sex and TNM stage, confirmed that *DLX6-AS1* methylation was an independent prognostic biomarker for poorer DSS (HR = 2.52, 95% CI: 1.35-4.69, *P* = 0.004, **Figure 6B**). This unfavorable effect was also identified in most individual CpG units, among which CpG_22 was the strongest indicator both in univariate regression model (HR = 2.67, 95% CI: 1.45-4.92, P = 0.002) (**Figure 6C**) and multivariate regression model (HR = 3.97, 95% CI: 2.08-7.57, P < 0.001) (**Figure 6D**). External validation based on additional 379 CRC patients (1.86 years of median follow-up time, 86 overall deaths) from the TCGA database confirmed the poorer prognosis with hypermethylated *DLX6-AS1* (*P* = 0.007), and the HR (95%CI) for overall survival (OS) by multivariate Cox proportional hazards regression model was 1.64 (1.02-2.64, *P *= 0.042) (**Supplementary Figure S10**).

### Construction of a Nomogram Model to Predict the Survival

**Figure 7:**
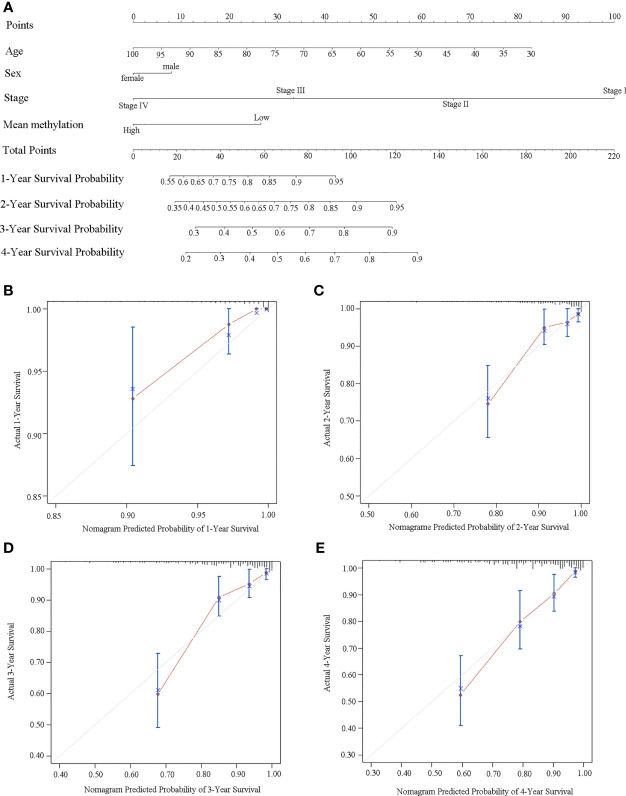
Establishment of a nomogram for survival prediction in our dataset. **(A)** Nomogram to predict the 1-, 2-, 3-, and 4-year survival of CRC patients. Calibration curves of **(B)** 1-year, **(C)** 2-year, **(D)** 3-year, and **(E)** 4-year survival nomogram model. The gray line represents the ideal predictive model, and the red line represents the observed model.

We further built a nomogram, including the methylation status of *DLX6-AS1* and clinical factors (age, gender, and TNM stage). The nomogram served as an individual’s prognostic predictor to predict the probability of disease-specific survival with 1-, 2-, 3-, and 4-year for CRC patients (**Figure 7A**). The C-index of the nomogram for predicting the DSS of CRC patients was 0.789 (95%CI: 0.681-0.897), and calibration curves for the 1-, 2-, 3-, and 4-year survival probability demonstrated optimal agreement between the prediction and actual observation (**Figures 7B–E**). Similar results were observed in the TCGA dataset (**Supplementary Figures S11**).

## Discussion

In this study, we performed a comprehensive DNA methylation profiling of lncRNAs in CRC and identified the novel methylated lncRNA, *DLX6-AS1*, as a promising biomarker. We validated the hypermethylation of *DLX6-AS1* in CRC, and further elucidated that the hypermethylation occurred since the NAA stage during multiple steps of the adenoma-carcinoma sequence. Further comparisons revealed that *DLX6-AS1* methylation was able to differentiate between CRC vs. NAA and AA vs. NAA. Moreover, the *DLX6-AS1* promoter hypermethylation was also identified in cfDNA of CRC patients as compared to healthy controls. Finally, survival analysis demonstrated *DLX6-AS1* hypermethylation as an independent predictor of poorer DSS and OS for CRC patients, and nomograms were constructed to predict the survival probability of individual CRC patients.

Most sporadic CRCs develop from dysplastic adenomas over a long time (2). This provides a desirable opportunity to detect CRC at an early curable stage and to screen for potentially premalignant lesions (23). Aberrant DNA promoter methylation has previously been revealed to be an early event in CRC development (24). For example, by conducting a series of genome-wide DNA methylation assays among 20 normal and pre-CRC samples, including 18 low-grade adenomas and 22 high-grade adenomas, Fan et al. found that the methylation alterations detected in low-grade adenoma were maintained or increased in high-grade adenoma and cancer (25). Several studies on DNA methylation biomarkers tested in fecal (26, 27) and blood (28, 29) samples indicated the potential of epigenetic biomarkers for early CRC diagnosis. The present study showed that *DLX6-AS1* hypermethylation was detectable since the NAA stage during colorectal neoplastic progression, suggesting that this epigenetic change is a candidate driver of tumor progression. Thus, *DLX6-AS1* hypermethylation might be a promising biomarker for the early detection and risk assessment of CRC.

It should be kept in mind that different histological adenomas differ in the risk of colorectal neoplastic progression (30). Based on a prospective cohort study, Click *et al.* revealed that patients with AA carried a higher risk of developing CRC than patients with NAA (31). To date, molecularly defined colorectal adenomas at high risk of progressing to CRC are limited (32). At the epigenetic level, Semaan *et al.* identified varied differences in *SEPT9* and *SHOX2* methylation levels among CRC, AA and NAA tissues (10). The present study revealed significant differences in *DLX6-AS1* methylation levels between CRC vs. NAA and AA vs. NAA. However, no significant differences in methylation levels were identified between AA and CRC, thus indicating that the biological processes inherent to CRC might probably be more active in AA than in NAA. These epigenetic features might be used to help characterize patients at a high risk for malignancy in the future.

Growing efforts have been made to identify noninvasive biomarkers for the early detection of CRC (33–35). Based on peripheral blood, Heiss et al. (36) reported the leukocyte DNA methylation of *KIAA1549L* and the leukocyte DNA methylation of *BCL2* as potential biomarkers for early CRC diagnosis. In the present study, we did not find significant differences in peripheral blood-based *DLX6-AS1* methylation levels between CRC patients, adenoma patients and healthy controls. Thus, the potential of this methylation marker in peripheral blood for early diagnosis requires further investigation. In fact, it remains controversial whether the DNA methylation alterations in peripheral blood are actually a response of the hematopoietic systems to tumor development (37). Another point of controversy to mention is whether the DNA methylation status measured in peripheral blood leukocytes could reflect the methylation status of local tumor lesions (38). To address this controversy, we then compared the methylation levels of *DLX6-AS1* between matched peripheral blood and local lesions. However, the lack of a correlation between them in the present study provides little evidence for the tissue origin of leukocyte methylation. These results indicate a distinct tissue-specific pattern of DNA methylation in CRC. As cfDNA is tumor derived and carries cancer-specific genetic and epigenetic aberrations (28, 39), we then observed the *DLX6-AS1* hypermethylation in the cfDNA samples from CRC patients as compared to healthy controls. Altogether, the methylation changes identified in our study might suggest a potential target for the study of cfDNA methylation for early cancer detection and tissue-of-origin mapping for metastases.

In clinical practice, CRC patient prognosis relies mostly on pathological staging according to the TNM system (40, 41). However, there are considerable variations in survival among individuals with the same staging (42), underlining the need for additional prognostic and predictive molecular markers. Here, we identified that *DLX6-AS1* methylation was associated with CRC-specific survival. Importantly, the identified methylation signature was independent of classical prognostic risk factors and could therefore be of added value when implemented in the clinic. *DLX6-AS1* was reported to participate in tumor progression independently or interactively with different targets (43, 44). For instance, Zhang et al. reported that *DLX6-AS1* promotes CRC cell proliferation, invasion and migration by modulating the PI3K/AKT/mTOR pathway (43). Another study carried out by Wang et al. suggested that *DLX6-AS1* plays an oncogenic role in bladder cancer through the miR-195-5p-mediated VEGFA/Ras/Raf/MEK/ERK pathway (44). More recently, Liang et al. revealed that high *DLX6-AS1* expression was associated with the poor clinical prognosis of gastric cancer (45), indicating its potential roles in cancer prognosis. Our study showed *DLX6-AS1* methylation to be associated with CRC-specific survival for the first time. Besides, the nomogram was generated to predict the survival probability of individual CRC patients and the calibration plots indicated that the predicted survival was consistent with the observed survival.The findings from this study indicate the potential importance of DNA methylation in CRC prognosis and provide clues to help improve clinical decision-making precision in the future.

We are aware of several limitations of this study. First, a direct explanation for the associations between DNA methylation and gene expression were limited since we are currently unable to measure the matched *DLX6-AS1* expression levels. Second, although hypermethylation of *DLX6-AS1* was observed in cfDNA samples by the 850K array in GEO database, further studies are needed taking into consideration of the low proportion of circulating tumor DNA in cfDNA and the currently very limited sample size. Third, as the follow-up in our study was relatively short, studies with longer clinical surveillance are warranted to bolster the reliability of the identified potential prognostic methylation biomarker. Last, although we found that the aberrant methylation of *DLX6-AS1* might serve as a potential biomarker for CRC progression and prognosis, external validation with larger and diverse study populations is still required to further confirm the clinical value of *DLX6-AS1* methylation in CRC.

In summary, based on a systematic evaluation of the DNA methylation pattern of lncRNAs in CRCs by genome-wide methylation profiling, the current study is the first to identify that the promoter region of *DLX6-AS1* was hypermethylated in CRC and its premalignant lesions. We additionally revealed that hypermethylation was independently associated with poorer DSS and OS in CRC patients. Thus, *DLX6-AS1* hypermethylation might occur at an early stage during colorectal carcinogenesis and has the potential to be a biomarker for the progression and prognosis of CRC.

## Data Availability Statement

The datasets presented in this study can be found in online repositories. The names of the repository/repositories and accession number(s) can be found in the article/**Supplementary Material**.

## Ethics Statement

The studies involving human participants were reviewed and approved by the Medical Ethics Committee of Zhejiang University School of Medicine. The patients/participants provided their written informed consent to participate in this study.

## Author Contributions

SL, MJ, and KC conceived and designed the study. SG, SQ, and YL performed the experiments and analyzed the data. JS, QL, JY, XY, ZL, MT, and JW recruited the participants and performed the histopathological evaluation. SL completed the manuscript with intellectual input from MJ and KC. All authors read and approved the final manuscript.

## Funding

This study was funded by the National Science Foundation of China (NSFC No. 81673262) and the National Basic Research Program of China (973 Program No. 2015CB554003).

## Conflict of Interest

The authors declare that the research was conducted in the absence of any commercial or financial relationships that could be construed as a potential conflict of interest.

## Publisher’s Note

All claims expressed in this article are solely those of the authors and do not necessarily represent those of their affiliated organizations, or those of the publisher, the editors and the reviewers. Any product that may be evaluated in this article, or claim that may be made by its manufacturer, is not guaranteed or endorsed by the publisher.

## Abbreviations

450K array, Illumina HumanMethylation450 BeadChip; 850K array, Illumina MethylationEPIC BeadChip; 95% CI, 95% confidence interval; AA, Advanced adenoma; AJCC, American Joint Committee on Cancer; ANOVA, Analysis of variance; AUC, Area under the curve; cfDNA, Cell-free DNA; C-index, Harrell’s concordance index; CRC, Colorectal cancer; DSS, Disease-specific survival; GEO, Gene Expression Omnibus; FAP, Familial adenomatous polyposis; HR, Hazard ratio; LncRNA, Long noncoding RNA; NAA, Nonadvanced adenoma; OS, Overall survival; ROC, Receiver operating characteristic; SD, Standard deviation; TSS, Transcription start site; TCGA, The Cancer Genome Atlas.
