# Supplementary material for: Genome-Wide Methylation Profiling of lncRNAs Reveals a Novel Progression-Related and Prognostic Marker for Colorectal Cancer
Source: Front Oncol. 2022 Jan 20;11:782077. doi: 10.3389/fonc.2021.782077 (PMC8811200; doi:10.3389/fonc.2021.782077)
Supplement: Supplementary file 1 [file DataSheet_1.docx]

Supplementary Material

**1. Supplementary Methods**

**Quality control and data processing of DNA methylation microarray**

Data by the Illumina HumanMethylation450 BeadChip methylation platform were processed using the ChAMP package in R software. Firstly, probes on X and Y chromosomes, those overlapped with known single-nucleotide polymorphisms, and those with cross-reactivation were excluded. And then those probes with low bead count (<3 beads in more than 5% of the samples) and low quality (as reported by detection values of *P* > 0.01) were further excluded. Secondly, methylation scores of each CpG site were represented as beta values ranging from 0 (non-methylated) to 1 (fully methylated), which were calculated by determining the ratios of methylated signal intensities to the sums of the methylated and unmethylated signal outputs. Then, the beta values were normalized using the beta mixture quartile method (BMIQ function). The batch effects were assessed using the singular value decomposition (SVD) method, and were corrected using ComBat function if the batch effects had statistical significance.

**2. Supplementary Tables**

| **Table S1.** Overview of the external datasets used in this study | | | |
| --- | --- | --- | --- |
| Dataset | Sample type | Source | Number of samples |
| TCGA dataset | Tissue | TCGA CRC | CRC = 393, Normal = 45 |
| GEO dataset | Tissue | GSE101764 | CRC = 104, Normal = 104 |
| GEO dataset | cfDNA | GSE122126 | CRC = 3, Normal = 4 |

| **Table S2.** Basic characteristics of the study participants subjected to blood-based analysis | | | | | |
| --- | --- | --- | --- | --- | --- |
| Characteristic | CRC (n = 60) | | Adenoma (n = 60) | | Control (n = 60) |
| Age, mean ± SD | 63.13 ± 9.63 | | 62.60 ± 6.94 | | 63.00 ± 9.62 |
| Sex, n (%) |  |  |  |  |  |
| Male | 35 (58.33) |  | 27 (45.00) |  | 35 (58.33) |
| Female | 25 (41.67) |  | 33 (55.00) |  | 25 (41.67) |
| Location, n (%) |  |  |  |  |  |
| Colon | 28 (46.67) |  | 43 (71.67) |  |  |
| Rectum | 32 (53.33) |  | 17 (28.33) |  |  |
| Stage, n (%) |  |  |  |  |  |
| I | 13 (21.67) |  |  |  |  |
| II | 13 (21.67) |  |  |  |  |
| III | 18 (30.00) |  |  |  |  |
| IV | 16 (26.67) |  |  |  |  |
| CRC, colorectal cancer; SD, standard deviation. | | | | | |

| **Table S3.** List of primer sequences used in the present study | | |
| --- | --- | --- |
| Gene | Primer | Sequence |
| *DLX6-AS1* | Forward | aggaagagag GAGGATTTGGTTTTTTTTAGGAGAG |
|  | Reverse | cagtaatacgactcactatagggagaaggct CCCAAAACCCAATAAAAATTACCT |
| *lnc-DPH5-1* | Forward | aggaagagag TGGGTTTTTTAGTAGTTTAGAGATGT |
|  | Reverse | cagtaatacgactcactatagggagaaggct CCCAAATCTTTCCTAAACAATAC |
| *lnc-PRSS2-6* | Forward | aggaagagag TTTGTTTATTTTGGGGATTTAGTTT |
|  | Reverse | cagtaatacgactcactatagggagaaggct ACTATAAACCAAAAACATTCAATACTTC |
| *lnc-RPS12-6* | Forward | aggaagagag TTAGTAGGTTTGGAAGGAGAGAAAA |
|  | Reverse | cagtaatacgactcactatagggagaaggct TCTAATAACCCCCTAAATAATCCAAA |
| *lnc-SFRP4-2* | Forward | aggaagagag TGGGATTTTGGAGTTAAGATTTTTAT |
|  | Reverse | cagtaatacgactcactatagggagaaggct AAATTCTATATTTCCTCTTCCTCAAAA |
| *SOX21-AS1* | Forward | aggaagagag GTTGGGGGATTAGTTTAGGGTTA |
|  | Reverse | cagtaatacgactcactatagggagaaggct CCCCCATCTACTTCTATCAAATAAA |

| **Table S4**. A list of differentially methylated lncRNAs identified in our dataset, TCGA dataset and GEO dataset | | | | | | | | | | | | | | | | | | |  |
| --- | --- | --- | --- | --- | --- | --- | --- | --- | --- | --- | --- | --- | --- | --- | --- | --- | --- | --- | --- |
| CpG ID | CHR | Location |  | Our dataset (850K array) | | | | |  | TCGA (450K array) | | | |  | GEO (450K array) | | | | |
|  |  |  |  | Methylation level | | | | |  | Methylation level | | | |  | Methylation level | | | | |
|  |  |  |  | lncRNA information | Tumor | NAT | Δβ | *P_adj_* |  | Tumor | NAT | Δβ | *P_adj_* |  | Tumor | NAT | Δβ | *P_adj_* | |
| cg00159100 | 7 | 37500000 |  | lnc-SFRP4-2 | 0.79 | 0.05 | 0.74 | 7.900E-07 |  | 0.50 | 0.07 | 0.43 | 9.421E-26 |  | 0.40 | 0.07 | 0.34 | 4.835E-31 | |
| cg10471437 | 16 | 28100000 |  | lnc-XPO6-1 | 0.79 | 0.06 | 0.73 | 7.900E-07 |  | 0.53 | 0.04 | 0.50 | 6.414E-32 |  | 0.46 | 0.05 | 0.41 | 2.931E-38 | |
| cg05807690^*^ | 2 | 101034283 |  | lnc-LONRF2-1 | 0.83 | 0.13 | 0.70 | 8.630E-06 |  | 0.49 | 0.17 | 0.31 | 1.614E-26 |  | 0.43 | 0.20 | 0.23 | 3.866E-31 | |
| cg21478136^*^ | 7 | 43200000 |  | lnc-PSMA2-1 | 0.83 | 0.13 | 0.70 | 1.270E-06 |  | 0.46 | 0.22 | 0.25 | 9.039E-51 |  | 0.43 | 0.21 | 0.21 | 2.059E-45 | |
| cg00995327 | 3 | 142838847 |  | lnc-SLC9A9-3 | 0.73 | 0.03 | 0.70 | 6.570E-06 |  | 0.47 | 0.06 | 0.41 | 4.671E-32 |  | 0.38 | 0.05 | 0.33 | 3.771E-32 | |
| cg17892556 | 19 | 12300000 |  | lnc-ZNF625-ZNF20-1 | 0.82 | 0.12 | 0.70 | 7.900E-07 |  | 0.64 | 0.09 | 0.55 | 7.449E-56 |  | 0.54 | 0.09 | 0.45 | 9.057E-52 | |
| cg06159352 | 7 | 37500000 |  | lnc-SFRP4-2 | 0.80 | 0.11 | 0.70 | 8.930E-07 |  | 0.58 | 0.12 | 0.46 | 5.931E-35 |  | 0.48 | 0.11 | 0.37 | 1.810E-38 | |
| cg08193528^*^ | 4 | 110223999 |  | lnc-SEC24B-1 | 0.81 | 0.11 | 0.69 | 3.250E-06 |  | 0.46 | 0.18 | 0.28 | 7.677E-49 |  | 0.41 | 0.18 | 0.23 | 2.102E-51 | |
| cg09638407 | 3 | 142839022 |  | lnc-SLC9A9-3 | 0.75 | 0.06 | 0.69 | 1.620E-06 |  | 0.51 | 0.09 | 0.42 | 1.587E-32 |  | 0.43 | 0.08 | 0.35 | 5.312E-34 | |
| cg00549566 | 7 | 37488253 |  | lnc-SFRP4-2 | 0.77 | 0.08 | 0.69 | 2.830E-06 |  | 0.51 | 0.10 | 0.41 | 7.950E-27 |  | 0.41 | 0.09 | 0.33 | 2.722E-29 | |
| cg02866454 | 10 | 7453455 |  | lnc-ITIH2-9 | 0.80 | 0.12 | 0.68 | 5.740E-07 |  | 0.63 | 0.15 | 0.47 | 1.863E-47 |  | 0.54 | 0.17 | 0.38 | 3.171E-45 | |
| cg26490272^*^ | 8 | 72756153 |  | lnc-TRPA1-2 | 0.78 | 0.11 | 0.68 | 6.570E-06 |  | 0.58 | 0.25 | 0.33 | 4.349E-54 |  | 0.49 | 0.22 | 0.27 | 3.360E-47 | |
| cg16818993 | 10 | 7453471 |  | lnc-ITIH2-9 | 0.73 | 0.05 | 0.68 | 3.450E-06 |  | 0.53 | 0.14 | 0.39 | 1.105E-41 |  | 0.44 | 0.08 | 0.36 | 7.282E-42 | |
| cg03853987 | 2 | 101034279 |  | lnc-LONRF2-1 | 0.82 | 0.15 | 0.68 | 8.760E-06 |  | 0.60 | 0.12 | 0.48 | 5.859E-26 |  | 0.47 | 0.11 | 0.37 | 5.261E-29 | |
| cg03535663 | 15 | 48937856 |  | lnc-EID1-4 | 0.74 | 0.06 | 0.68 | 2.340E-06 |  | 0.47 | 0.07 | 0.41 | 1.081E-27 |  | 0.40 | 0.07 | 0.34 | 1.295E-38 | |
| cg12584684 | 4 | 96471105 |  | lnc-PDHA2-2 | 0.76 | 0.09 | 0.67 | 8.390E-06 |  | 0.58 | 0.08 | 0.50 | 9.018E-41 |  | 0.48 | 0.07 | 0.40 | 5.408E-40 | |
| cg03921753 | 16 | 28074670 |  | lnc-XPO6-1 | 0.77 | 0.10 | 0.67 | 1.620E-06 |  | 0.51 | 0.09 | 0.42 | 9.338E-42 |  | 0.41 | 0.08 | 0.33 | 4.770E-38 | |
| cg26878816 | 10 | 7452598 |  | lnc-ITIH2-9 | 0.70 | 0.04 | 0.67 | 2.110E-06 |  | 0.54 | 0.10 | 0.44 | 6.281E-42 |  | 0.48 | 0.07 | 0.40 | 1.551E-46 | |
| cg07334393^*^ | 1 | 109000000 |  | VAV3-AS1 | 0.72 | 0.06 | 0.66 | 1.270E-06 |  | 0.47 | 0.21 | 0.26 | 8.170E-39 |  | 0.42 | 0.22 | 0.20 | 6.006E-36 | |
| cg03394150 | 16 | 28074384 |  | lnc-XPO6-1 | 0.75 | 0.10 | 0.66 | 3.180E-06 |  | 0.61 | 0.03 | 0.58 | 9.772E-52 |  | 0.53 | 0.07 | 0.47 | 5.843E-50 | |
| cg12701184^*^ | 12 | 95942212 |  | lnc-NTN4-3 | 0.75 | 0.09 | 0.66 | 7.480E-06 |  | 0.58 | 0.20 | 0.38 | 1.307E-45 |  | 0.50 | 0.21 | 0.28 | 4.764E-39 | |
| cg07279933 | 6 | 127440413 |  | lnc-ECHDC1-1 | 0.71 | 0.06 | 0.65 | 7.010E-06 |  | 0.47 | 0.07 | 0.40 | 8.183E-23 |  | 0.38 | 0.07 | 0.31 | 4.538E-28 | |
| cg09493063 | 7 | 142494985 |  | lnc-PRSS2-6 | 0.83 | 0.18 | 0.65 | 4.910E-06 |  | 0.66 | 0.15 | 0.52 | 2.415E-60 |  | 0.57 | 0.12 | 0.46 | 3.326E-46 | |
| cg23749856 | 3 | 142839837 |  | lnc-SLC9A9-3 | 0.74 | 0.09 | 0.65 | 3.990E-06 |  | 0.60 | 0.13 | 0.47 | 5.388E-58 |  | 0.53 | 0.10 | 0.43 | 2.546E-57 | |
| cg11855526 | 11 | 30607068 |  | MPPED2-AS1 | 0.76 | 0.11 | 0.65 | 2.500E-06 |  | 0.50 | 0.03 | 0.47 | 1.032E-22 |  | 0.40 | 0.06 | 0.34 | 3.793E-27 | |
| cg20150591 | 7 | 3341694 |  | lnc-CARD11-8 | 0.79 | 0.14 | 0.65 | 1.640E-06 |  | 0.61 | 0.11 | 0.50 | 6.530E-34 |  | 0.48 | 0.11 | 0.37 | 8.415E-35 | |
| cg24662718 | 1 | 108507468 |  | VAV3-AS1 | 0.76 | 0.12 | 0.65 | 1.640E-06 |  | 0.52 | 0.14 | 0.38 | 1.501E-27 |  | 0.43 | 0.09 | 0.34 | 5.991E-32 | |
| cg05062333 | 6 | 133562269 |  | lnc-RPS12-6 | 0.77 | 0.12 | 0.64 | 3.450E-06 |  | 0.53 | 0.11 | 0.42 | 5.183E-44 |  | 0.45 | 0.10 | 0.36 | 3.156E-40 | |
| cg00501272 | 6 | 29760505 |  | lnc-ZFP57-16 | 0.73 | 0.09 | 0.64 | 9.220E-06 |  | 0.57 | 0.21 | 0.36 | 3.085E-40 |  | 0.51 | 0.15 | 0.35 | 1.760E-46 | |
| cg25744484^*^ | 8 | 104512877 |  | lnc-SLC25A32-3 | 0.80 | 0.16 | 0.64 | 3.540E-06 |  | 0.37 | 0.15 | 0.22 | 6.734E-31 |  | 0.32 | 0.12 | 0.21 | 3.855E-35 | |
| cg07523553 | 11 | 1770066 |  | lnc-CTSD-3 | 0.80 | 0.16 | 0.64 | 1.620E-06 |  | 0.53 | 0.14 | 0.39 | 2.070E-34 |  | 0.44 | 0.10 | 0.33 | 9.066E-36 | |
| cg24176563 | 6 | 133562776 |  | lnc-RPS12-6 | 0.83 | 0.19 | 0.64 | 3.490E-06 |  | 0.55 | 0.20 | 0.36 | 1.067E-50 |  | 0.50 | 0.14 | 0.37 | 8.783E-50 | |
| cg12621171 | 5 | 88185768 |  | MEF2C-AS1 | 0.73 | 0.10 | 0.63 | 3.250E-06 |  | 0.48 | 0.08 | 0.39 | 7.380E-32 |  | 0.38 | 0.07 | 0.31 | 1.198E-33 | |
| cg14270292 | 6 | 133562494 |  | lnc-RPS12-6 | 0.77 | 0.14 | 0.63 | 6.270E-06 |  | 0.59 | 0.09 | 0.50 | 1.648E-56 |  | 0.50 | 0.09 | 0.40 | 1.976E-46 | |
| cg10571951 | 5 | 88185387 |  | MEF2C-AS1 | 0.68 | 0.05 | 0.63 | 7.250E-06 |  | 0.44 | 0.07 | 0.37 | 1.133E-25 |  | 0.31 | 0.05 | 0.26 | 1.134E-26 | |
| cg08430489 | 7 | 142494953 |  | lnc-PRSS2-6 | 0.74 | 0.12 | 0.63 | 7.110E-06 |  | 0.58 | 0.07 | 0.52 | 1.183E-55 |  | 0.50 | 0.09 | 0.40 | 6.523E-47 | |
| cg09734791 | 8 | 72756155 |  | lnc-TRPA1-2 | 0.73 | 0.10 | 0.62 | 7.340E-06 |  | 0.59 | 0.09 | 0.51 | 6.167E-72 |  | 0.54 | 0.10 | 0.44 | 1.803E-63 | |
| cg20506184 | 10 | 7453329 |  | lnc-ITIH2-9 | 0.71 | 0.09 | 0.62 | 6.940E-06 |  | 0.56 | 0.19 | 0.37 | 6.462E-34 |  | 0.48 | 0.12 | 0.36 | 2.638E-42 | |
| cg08774368 | 3 | 142839578 |  | lnc-SLC9A9-3 | 0.69 | 0.07 | 0.62 | 3.790E-06 |  | 0.54 | 0.03 | 0.51 | 3.568E-61 |  | 0.50 | 0.07 | 0.43 | 1.544E-53 | |
| cg06269753 | 8 | 72755871 |  | lnc-TRPA1-2 | 0.81 | 0.19 | 0.62 | 5.780E-06 |  | 0.59 | 0.22 | 0.37 | 1.369E-47 |  | 0.49 | 0.14 | 0.35 | 1.893E-48 | |
| cg13777681 | 2 | 115920221 |  | DPP10-AS1 | 0.82 | 0.20 | 0.62 | 1.620E-06 |  | 0.59 | 0.18 | 0.41 | 1.348E-35 |  | 0.50 | 0.14 | 0.36 | 2.035E-40 | |
| cg11202584^*^ | 3 | 143000000 |  | lnc-SLC9A9-3 | 0.74 | 0.12 | 0.62 | 1.370E-06 |  | 0.54 | 0.25 | 0.29 | 4.135E-48 |  | 0.49 | 0.25 | 0.23 | 7.760E-46 | |
| cg16741041 | 13 | 36920332 |  | SPART-AS1 | 0.77 | 0.16 | 0.61 | 4.790E-06 |  | 0.58 | 0.05 | 0.53 | 6.833E-44 |  | 0.50 | 0.11 | 0.39 | 4.128E-40 | |
| cg27347269 | 18 | 67067867 |  | lnc-CD226-8 | 0.74 | 0.13 | 0.61 | 6.340E-06 |  | 0.57 | 0.06 | 0.51 | 9.867E-54 |  | 0.48 | 0.08 | 0.40 | 3.294E-41 | |
| cg05194233^*^ | 5 | 128000000 |  | lnc-C5orf63-6 | 0.78 | 0.16 | 0.61 | 1.370E-06 |  | 0.56 | 0.21 | 0.34 | 2.836E-45 |  | 0.49 | 0.18 | 0.31 | 4.944E-41 | |
| cg19283506 | 2 | 101034270 |  | lnc-LONRF2-1 | 0.82 | 0.21 | 0.61 | 5.140E-06 |  | 0.58 | 0.10 | 0.48 | 6.986E-22 |  | 0.49 | 0.14 | 0.36 | 6.190E-28 | |
| cg26449787 | 6 | 133562087 |  | lnc-RPS12-6 | 0.76 | 0.15 | 0.61 | 7.190E-06 |  | 0.56 | 0.11 | 0.45 | 2.380E-50 |  | 0.47 | 0.10 | 0.37 | 1.231E-42 | |
| cg13916740 | 19 | 56904997 |  | ZNF582-AS1 | 0.74 | 0.13 | 0.61 | 6.910E-06 |  | 0.50 | 0.04 | 0.46 | 5.378E-31 |  | 0.47 | 0.06 | 0.41 | 5.876E-43 | |
| cg15737840^*^ | 1 | 108507483 |  | VAV3-AS1 | 0.72 | 0.11 | 0.61 | 3.370E-06 |  | 0.47 | 0.21 | 0.26 | 8.170E-39 |  | 0.42 | 0.22 | 0.20 | 6.006E-36 | |
| cg20286200 | 6 | 133562267 |  | lnc-RPS12-6 | 0.73 | 0.12 | 0.61 | 2.190E-06 |  | 0.58 | 0.09 | 0.48 | 2.049E-57 |  | 0.49 | 0.09 | 0.39 | 7.422E-43 | |
| cg22143569 | 6 | 11044541 |  | ELOVL2-AS1 | 0.67 | 0.06 | 0.60 | 4.930E-06 |  | 0.43 | 0.03 | 0.40 | 7.190E-23 |  | 0.36 | 0.05 | 0.32 | 4.358E-31 | |
| cg01950845 | 15 | 93632730 |  | lnc-CHD2-2 | 0.70 | 0.10 | 0.60 | 4.710E-06 |  | 0.46 | 0.04 | 0.42 | 1.180E-24 |  | 0.41 | 0.06 | 0.35 | 2.047E-31 | |
| cg21621906 | 7 | 142494914 |  | lnc-PRSS2-6 | 0.65 | 0.05 | 0.60 | 8.750E-06 |  | 0.58 | 0.04 | 0.55 | 3.765E-51 |  | 0.50 | 0.06 | 0.44 | 6.482E-48 | |
| cg10539069 | 7 | 93520275 |  | lnc-GNG11-1 | 0.73 | 0.12 | 0.60 | 8.820E-06 |  | 0.54 | 0.23 | 0.31 | 9.569E-29 |  | 0.43 | 0.13 | 0.30 | 3.240E-32 | |
| cg11965976 | 5 | 127874463 |  | lnc-C5orf63-6 | 0.79 | 0.19 | 0.60 | 4.120E-06 |  | 0.63 | 0.14 | 0.49 | 2.859E-38 |  | 0.50 | 0.13 | 0.37 | 6.470E-32 | |
| cg14443519 | 6 | 29760410 |  | lnc-ZFP57-16 | 0.72 | 0.12 | 0.60 | 7.640E-06 |  | 0.63 | 0.18 | 0.44 | 8.450E-51 |  | 0.57 | 0.17 | 0.40 | 1.403E-50 | |
| cg10480343 | 15 | 48938347 |  | lnc-EID1-4 | 0.68 | 0.08 | 0.60 | 7.140E-06 |  | 0.47 | 0.03 | 0.44 | 2.451E-28 |  | 0.43 | 0.07 | 0.36 | 4.620E-36 | |
| cg04741853 | 3 | 44037189 |  | lnc-TOPAZ1-7 | 0.73 | 0.14 | 0.60 | 7.660E-06 |  | 0.52 | 0.14 | 0.38 | 2.589E-27 |  | 0.47 | 0.15 | 0.32 | 6.545E-31 | |
| cg13879483 | 12 | 95942907 |  | lnc-NTN4-3 | 0.81 | 0.22 | 0.60 | 6.030E-06 |  | 0.76 | 0.13 | 0.63 | 7.310E-55 |  | 0.60 | 0.19 | 0.41 | 1.582E-38 | |
| cg12973930 | 19 | 57049695 |  | lnc-ZNF667-3 | 0.77 | 0.17 | 0.60 | 4.310E-06 |  | 0.38 | 0.08 | 0.30 | 3.669E-13 |  | 0.38 | 0.08 | 0.29 | 1.126E-23 | |
| cg09275869 | 18 | 44337099 |  | lnc-KATNAL2-1 | 0.80 | 0.21 | 0.59 | 8.410E-06 |  | 0.56 | 0.17 | 0.39 | 9.546E-35 |  | 0.47 | 0.14 | 0.33 | 3.270E-35 | |
| cg21479226 | 8 | 69244510 |  | C8orf34-AS1 | 0.81 | 0.21 | 0.59 | 7.970E-06 |  | 0.76 | 0.13 | 0.63 | 2.385E-71 |  | 0.64 | 0.16 | 0.48 | 3.010E-50 | |
| cg25848557 | 1 | 108507766 |  | VAV3-AS1 | 0.65 | 0.06 | 0.59 | 2.000E-06 |  | 0.40 | 0.02 | 0.38 | 2.326E-25 |  | 0.36 | 0.05 | 0.31 | 8.175E-31 | |
| cg14287112 | 6 | 133562485 |  | lnc-RPS12-6 | 0.72 | 0.13 | 0.59 | 7.660E-06 |  | 0.58 | 0.05 | 0.53 | 2.108E-55 |  | 0.52 | 0.10 | 0.41 | 4.552E-49 | |
| cg11901272 | 6 | 29760447 |  | lnc-ZFP57-16 | 0.71 | 0.13 | 0.59 | 4.190E-06 |  | 0.53 | 0.23 | 0.30 | 3.302E-35 |  | 0.48 | 0.16 | 0.32 | 7.508E-47 | |
| cg01162672 | 6 | 133562275 |  | lnc-RPS12-6 | 0.77 | 0.18 | 0.59 | 8.090E-06 |  | 0.50 | 0.14 | 0.36 | 1.595E-37 |  | 0.43 | 0.12 | 0.31 | 9.506E-41 | |
| cg24870497 | 4 | 96469519 |  | lnc-PDHA2-2 | 0.69 | 0.10 | 0.59 | 8.740E-06 |  | 0.44 | 0.06 | 0.38 | 9.520E-31 |  | 0.37 | 0.08 | 0.29 | 3.905E-38 | |
| cg22871668 | 6 | 133562492 |  | lnc-RPS12-6 | 0.76 | 0.18 | 0.59 | 6.570E-06 |  | 0.60 | 0.04 | 0.56 | 1.626E-58 |  | 0.52 | 0.10 | 0.43 | 4.812E-49 | |
| cg05446010 | 6 | 11044558 |  | ELOVL2-AS1 | 0.62 | 0.03 | 0.58 | 3.450E-06 |  | 0.34 | 0.02 | 0.32 | 4.790E-19 |  | 0.30 | 0.03 | 0.27 | 8.172E-28 | |
| cg23849169 | 6 | 29760495 |  | lnc-ZFP57-16 | 0.72 | 0.14 | 0.58 | 7.250E-06 |  | 0.55 | 0.25 | 0.30 | 4.523E-38 |  | 0.48 | 0.17 | 0.31 | 3.257E-45 | |
| cg15649801 | 7 | 93520269 |  | lnc-GNG11-1 | 0.70 | 0.11 | 0.58 | 8.760E-06 |  | 0.53 | 0.21 | 0.32 | 2.436E-29 |  | 0.43 | 0.12 | 0.31 | 4.138E-31 | |
| cg22535307 | 15 | 48938370 |  | lnc-EID1-4 | 0.71 | 0.13 | 0.58 | 7.630E-06 |  | 0.50 | 0.05 | 0.46 | 1.767E-33 |  | 0.45 | 0.08 | 0.36 | 1.485E-35 | |
| cg01939274 | 5 | 127874478 |  | lnc-C5orf63-6 | 0.78 | 0.20 | 0.58 | 3.990E-06 |  | 0.65 | 0.09 | 0.55 | 1.807E-33 |  | 0.52 | 0.14 | 0.38 | 7.228E-34 | |
| cg02246645 | 12 | 103352235 |  | lnc-PAH-3 | 0.63 | 0.05 | 0.58 | 3.250E-06 |  | 0.37 | 0.07 | 0.31 | 1.050E-19 |  | 0.33 | 0.06 | 0.27 | 1.583E-27 | |
| cg25532099 | 5 | 127874466 |  | lnc-C5orf63-6 | 0.83 | 0.25 | 0.58 | 2.830E-06 |  | 0.58 | 0.17 | 0.42 | 1.188E-31 |  | 0.49 | 0.13 | 0.36 | 6.210E-32 | |
| cg21045608 | 11 | 8615456 |  | lnc-TRIM66-6 | 0.65 | 0.08 | 0.57 | 6.340E-06 |  | 0.46 | 0.13 | 0.34 | 2.941E-18 |  | 0.42 | 0.09 | 0.33 | 3.291E-32 | |
| cg01328892 | 6 | 133562332 |  | lnc-RPS12-6 | 0.80 | 0.22 | 0.57 | 3.450E-06 |  | 0.62 | 0.16 | 0.46 | 1.423E-48 |  | 0.53 | 0.14 | 0.39 | 5.805E-39 | |
| cg08858437 | 3 | 142838938 |  | lnc-SLC9A9-3 | 0.65 | 0.08 | 0.57 | 4.720E-06 |  | 0.48 | 0.04 | 0.44 | 7.249E-33 |  | 0.42 | 0.08 | 0.34 | 1.514E-32 | |
| cg12767281 | 10 | 7452950 |  | lnc-ITIH2-9 | 0.70 | 0.13 | 0.57 | 7.150E-06 |  | 0.48 | 0.05 | 0.43 | 2.114E-31 |  | 0.44 | 0.10 | 0.34 | 1.702E-37 | |
| cg09528825 | 16 | 28074388 |  | lnc-XPO6-1 | 0.68 | 0.12 | 0.57 | 4.910E-06 |  | 0.55 | 0.04 | 0.51 | 1.087E-57 |  | 0.49 | 0.08 | 0.41 | 2.114E-51 | |
| cg17859110 | 20 | 41818770 |  | lnc-SRSF6-1 | 0.71 | 0.14 | 0.57 | 6.650E-06 |  | 0.62 | 0.06 | 0.57 | 2.750E-45 |  | 0.50 | 0.10 | 0.41 | 2.348E-37 | |
| cg17958315 | 5 | 127873552 |  | lnc-C5orf63-6 | 0.71 | 0.14 | 0.57 | 7.860E-06 |  | 0.41 | 0.03 | 0.38 | 3.734E-25 |  | 0.38 | 0.07 | 0.31 | 1.070E-34 | |
| cg26978668 | 10 | 7453452 |  | lnc-ITIH2-9 | 0.63 | 0.07 | 0.56 | 7.510E-06 |  | 0.49 | 0.17 | 0.32 | 6.388E-38 |  | 0.41 | 0.10 | 0.31 | 6.477E-46 | |
| cg11664500 | 6 | 133562479 |  | lnc-RPS12-6 | 0.74 | 0.18 | 0.56 | 7.150E-06 |  | 0.60 | 0.05 | 0.55 | 1.091E-59 |  | 0.53 | 0.12 | 0.41 | 8.783E-50 | |
| cg09410234 | 1 | 111217715 |  | lnc-KCNA3-3 | 0.79 | 0.24 | 0.56 | 8.860E-06 |  | 0.63 | 0.26 | 0.37 | 1.121E-44 |  | 0.52 | 0.18 | 0.34 | 6.953E-42 | |
| cg13807970 | 2 | 145274441 |  | lnc-ZEB2-1 | 0.65 | 0.10 | 0.55 | 4.720E-06 |  | 0.49 | 0.04 | 0.45 | 1.470E-39 |  | 0.46 | 0.08 | 0.37 | 5.992E-43 | |
| cg02667667 | 16 | 56228505 |  | lnc-AMFR-2 | 0.74 | 0.18 | 0.55 | 7.260E-06 |  | 0.54 | 0.07 | 0.47 | 4.992E-28 |  | 0.46 | 0.10 | 0.36 | 8.556E-34 | |
| cg21392309^*^ | 16 | 51185376 |  | lnc-HNRNPA1P48-124 | 0.73 | 0.17 | 0.55 | 8.760E-06 |  | 0.59 | 0.32 | 0.27 | 9.603E-51 |  | 0.52 | 0.27 | 0.25 | 3.160E-41 | |
| cg00512279 | 10 | 119000991 |  | lnc-PDZD8-1 | 0.80 | 0.25 | 0.55 | 7.660E-06 |  | 0.49 | 0.20 | 0.29 | 9.283E-24 |  | 0.40 | 0.15 | 0.25 | 3.288E-26 | |
| cg05109245 | 18 | 18822579 |  | lnc-ROCK1-2 | 0.67 | 0.12 | 0.55 | 8.430E-06 |  | 0.32 | 0.03 | 0.29 | 3.665E-13 |  | 0.29 | 0.07 | 0.22 | 1.083E-19 | |
| cg09926747 | 4 | 110224247 |  | lnc-SEC24B-1 | 0.76 | 0.22 | 0.55 | 9.220E-06 |  | 0.59 | 0.19 | 0.39 | 3.201E-47 |  | 0.49 | 0.15 | 0.34 | 1.405E-47 | |
| cg01907051 | 6 | 159591121 |  | lnc-TAGAP-4 | 0.86 | 0.31 | 0.55 | 7.510E-06 |  | 0.55 | 0.24 | 0.31 | 4.721E-29 |  | 0.49 | 0.18 | 0.32 | 7.036E-40 | |
| cg15248264^*^ | 10 | 83634082 |  | lnc-DYDC1-3 | 0.68 | 0.13 | 0.55 | 9.430E-06 |  | 0.50 | 0.23 | 0.27 | 8.116E-49 |  | 0.43 | 0.21 | 0.22 | 3.049E-39 | |
| cg07829842 | 16 | 56228495 |  | lnc-AMFR-2 | 0.72 | 0.17 | 0.55 | 8.810E-06 |  | 0.54 | 0.05 | 0.49 | 3.611E-33 |  | 0.46 | 0.10 | 0.36 | 5.146E-35 | |
| cg15531403 | 8 | 53852184 |  | lnc-RB1CC1-1 | 0.78 | 0.23 | 0.55 | 8.610E-06 |  | 0.67 | 0.16 | 0.51 | 1.461E-52 |  | 0.53 | 0.18 | 0.36 | 3.244E-43 | |
| cg17641861 | 4 | 570361 |  | lnc-PDE6B-1 | 0.76 | 0.21 | 0.54 | 9.940E-06 |  | 0.62 | 0.09 | 0.53 | 7.022E-47 |  | 0.51 | 0.13 | 0.38 | 1.607E-44 | |
| cg08439930 | 16 | 51185060 |  | lnc-HNRNPA1P48-124 | 0.83 | 0.28 | 0.54 | 8.340E-06 |  | 0.71 | 0.16 | 0.55 | 2.103E-54 |  | 0.58 | 0.19 | 0.38 | 5.671E-44 | |
| cg07095995 | 4 | 110223980 |  | lnc-SEC24B-1 | 0.74 | 0.20 | 0.54 | 7.390E-06 |  | 0.57 | 0.13 | 0.44 | 2.085E-55 |  | 0.48 | 0.11 | 0.37 | 2.048E-50 | |
| cg21607030 | 6 | 133563532 |  | lnc-RPS12-6 | 0.78 | 0.24 | 0.54 | 7.150E-06 |  | 0.64 | 0.12 | 0.52 | 1.568E-47 |  | 0.49 | 0.12 | 0.38 | 1.595E-39 | |
| cg12740527 | 11 | 8289974 |  | lnc-TUB-8 | 0.66 | 0.13 | 0.54 | 6.480E-06 |  | 0.46 | 0.03 | 0.43 | 1.437E-31 |  | 0.42 | 0.07 | 0.35 | 2.032E-35 | |
| cg09461837 | 7 | 3341524 |  | lnc-CARD11-8 | 0.65 | 0.11 | 0.54 | 6.480E-06 |  | 0.42 | 0.05 | 0.37 | 8.534E-32 |  | 0.38 | 0.09 | 0.29 | 1.920E-35 | |
| cg18023283 | 12 | 85306828 |  | lnc-LRRIQ1-1 | 0.84 | 0.30 | 0.54 | 6.940E-06 |  | 0.73 | 0.19 | 0.54 | 1.798E-56 |  | 0.63 | 0.21 | 0.42 | 4.202E-48 | |
| cg21510680^*^ | 1 | 108506979 |  | VAV3-AS1 | 0.71 | 0.18 | 0.54 | 8.400E-06 |  | 0.47 | 0.21 | 0.26 | 8.170E-39 |  | 0.42 | 0.22 | 0.20 | 6.006E-36 | |
| cg20924470 | 5 | 135265849 |  | lnc-SLC25A48-4 | 0.70 | 0.16 | 0.53 | 7.700E-06 |  | 0.55 | 0.14 | 0.41 | 5.460E-38 |  | 0.43 | 0.12 | 0.31 | 6.810E-37 | |
| cg27442308 | 13 | 95364675 |  | SOX21-AS1 | 0.74 | 0.21 | 0.53 | 9.120E-06 |  | 0.58 | 0.06 | 0.52 | 9.504E-49 |  | 0.51 | 0.12 | 0.38 | 1.381E-44 | |
| cg03333330 | 7 | 93520346 |  | lnc-GNG11-1 | 0.77 | 0.24 | 0.53 | 8.400E-06 |  | 0.63 | 0.32 | 0.30 | 7.701E-32 |  | 0.51 | 0.22 | 0.29 | 1.929E-31 | |
| cg17722675 | 6 | 133563382 |  | lnc-RPS12-6 | 0.81 | 0.28 | 0.53 | 7.150E-06 |  | 0.59 | 0.23 | 0.37 | 6.662E-53 |  | 0.52 | 0.18 | 0.34 | 2.043E-47 | |
| cg03976877 | 7 | 158937610 |  | lnc-VIPR2-1 | 0.81 | 0.28 | 0.53 | 5.010E-06 |  | 0.67 | 0.08 | 0.59 | 1.050E-58 |  | 0.58 | 0.15 | 0.43 | 3.531E-49 | |
| cg00246932 | 16 | 56228498 |  | lnc-AMFR-2 | 0.70 | 0.17 | 0.53 | 8.330E-06 |  | 0.57 | 0.06 | 0.51 | 2.540E-34 |  | 0.49 | 0.13 | 0.37 | 8.253E-37 | |
| cg18323466 | 1 | 101702602 |  | lnc-DPH5-1 | 0.65 | 0.12 | 0.53 | 7.860E-06 |  | 0.50 | 0.09 | 0.41 | 1.978E-19 |  | 0.43 | 0.13 | 0.30 | 5.227E-26 | |
| cg19721867 | 10 | 119001066 |  | lnc-PDZD8-1 | 0.60 | 0.07 | 0.53 | 8.210E-06 |  | 0.31 | 0.02 | 0.29 | 1.888E-16 |  | 0.27 | 0.06 | 0.22 | 7.595E-20 | |
| cg11278204 | 7 | 3341875 |  | lnc-CARD11-8 | 0.70 | 0.17 | 0.53 | 3.720E-06 |  | 0.50 | 0.16 | 0.34 | 1.386E-27 |  | 0.40 | 0.13 | 0.27 | 2.404E-31 | |
| cg01555431 | 6 | 151562026 |  | lnc-ZBTB2-5 | 0.70 | 0.18 | 0.53 | 7.530E-06 |  | 0.50 | 0.18 | 0.32 | 5.373E-19 |  | 0.42 | 0.12 | 0.30 | 1.628E-25 | |
| cg23860325 | 7 | 54271152 |  | lnc-SEC61G-14 | 0.23 | 0.76 | -0.52 | 6.720E-06 |  | 0.25 | 0.65 | -0.40 | 5.479E-76 |  | 0.35 | 0.72 | -0.37 | 1.092E-55 | |
| cg11518846 | 6 | 133562246 |  | lnc-RPS12-6 | 0.67 | 0.15 | 0.52 | 8.750E-06 |  | 0.49 | 0.04 | 0.44 | 2.844E-44 |  | 0.43 | 0.09 | 0.34 | 3.857E-40 | |
| cg00901051 | 1 | 208132490 |  | lnc-CD46-2 | 0.72 | 0.20 | 0.52 | 4.790E-06 |  | 0.55 | 0.14 | 0.40 | 2.538E-39 |  | 0.46 | 0.13 | 0.33 | 1.776E-38 | |
| cg27420520 | 12 | 103352267 |  | lnc-PAH-3 | 0.56 | 0.04 | 0.52 | 7.150E-06 |  | 0.36 | 0.03 | 0.33 | 4.331E-23 |  | 0.34 | 0.05 | 0.29 | 4.093E-31 | |
| cg23330563^*^ | 1 | 108506977 |  | VAV3-AS1 | 0.75 | 0.23 | 0.52 | 7.630E-06 |  | 0.47 | 0.21 | 0.26 | 8.170E-39 |  | 0.42 | 0.22 | 0.20 | 6.006E-36 | |
| cg10566121 | 11 | 30606026 |  | MPPED2-AS1 | 0.70 | 0.19 | 0.52 | 8.040E-06 |  | 0.63 | 0.09 | 0.55 | 6.877E-38 |  | 0.51 | 0.14 | 0.37 | 8.102E-39 | |
| cg01671881 | 20 | 31804901 |  | lnc-CDK5RAP1-1 | 0.24 | 0.75 | -0.51 | 5.690E-06 |  | 0.32 | 0.64 | -0.32 | 4.158E-41 |  | 0.36 | 0.71 | -0.34 | 2.445E-50 | |
| cg20980055 | 6 | 133562258 |  | lnc-RPS12-6 | 0.60 | 0.09 | 0.51 | 8.250E-06 |  | 0.45 | 0.04 | 0.41 | 1.552E-43 |  | 0.40 | 0.08 | 0.32 | 3.274E-42 | |
| cg08599259 | 1 | 38510933 |  | lnc-POU3F1-3 | 0.70 | 0.19 | 0.51 | 7.150E-06 |  | 0.48 | 0.10 | 0.37 | 8.231E-23 |  | 0.39 | 0.14 | 0.26 | 6.148E-28 | |
| cg10096177 | 12 | 103889516 |  | lnc-NT5DC3-5 | 0.60 | 0.09 | 0.51 | 6.480E-06 |  | 0.44 | 0.12 | 0.32 | 1.305E-16 |  | 0.39 | 0.10 | 0.29 | 1.283E-25 | |
| cg00177451 | 16 | 51186266 |  | lnc-HNRNPA1P48-124 | 0.67 | 0.16 | 0.51 | 9.990E-06 |  | 0.63 | 0.18 | 0.45 | 1.920E-47 |  | 0.52 | 0.16 | 0.37 | 1.864E-39 | |
| cg02446647 | 6 | 133562101 |  | lnc-RPS12-6 | 0.76 | 0.26 | 0.51 | 8.190E-06 |  | 0.68 | 0.23 | 0.44 | 1.367E-60 |  | 0.59 | 0.23 | 0.36 | 2.071E-50 | |
| cg15790037 | 17 | 37321490 |  | lnc-ARL5C-1 | 0.67 | 0.17 | 0.50 | 5.710E-06 |  | 0.62 | 0.30 | 0.32 | 2.101E-29 |  | 0.54 | 0.24 | 0.30 | 4.703E-34 | |
| cg04891086 | 12 | 5018513 |  | lnc-KCNA1-97 | 0.81 | 0.31 | 0.50 | 9.660E-06 |  | 0.64 | 0.26 | 0.38 | 1.231E-39 |  | 0.51 | 0.18 | 0.32 | 7.107E-37 | |
| cg23934404 | 13 | 112758491 |  | lnc-SOX1-5 | 0.74 | 0.24 | 0.50 | 3.450E-06 |  | 0.64 | 0.15 | 0.49 | 6.056E-54 |  | 0.53 | 0.18 | 0.35 | 1.854E-48 | |
| cg06429617 | 19 | 12300000 |  | lnc-ZNF625-ZNF20-1 | 0.60 | 0.10 | 0.50 | 1.590E-06 |  | 0.45 | 0.04 | 0.41 | 1.701E-39 |  | 0.41 | 0.08 | 0.33 | 8.522E-50 | |
| cg10236452 | 1 | 208132590 |  | lnc-CD46-2 | 0.62 | 0.13 | 0.49 | 6.030E-06 |  | 0.44 | 0.06 | 0.38 | 6.048E-36 |  | 0.40 | 0.09 | 0.31 | 1.127E-39 | |
| cg26014634 | 17 | 70113514 |  | lnc-SLC39A11-10 | 0.67 | 0.18 | 0.49 | 4.710E-06 |  | 0.50 | 0.27 | 0.23 | 3.010E-19 |  | 0.42 | 0.20 | 0.23 | 1.561E-30 | |
| cg04996873 | 1 | 14925992 |  | lnc-CASP9-8 | 0.83 | 0.34 | 0.49 | 5.380E-06 |  | 0.67 | 0.25 | 0.42 | 9.497E-75 |  | 0.57 | 0.24 | 0.34 | 7.060E-44 | |
| cg20787173 | 6 | 133562475 |  | lnc-RPS12-6 | 0.74 | 0.25 | 0.49 | 7.630E-06 |  | 0.65 | 0.16 | 0.49 | 1.933E-64 |  | 0.58 | 0.24 | 0.34 | 2.486E-50 | |
| cg20754261 | 2 | 468330 |  | lnc-ACP1-6 | 0.78 | 0.29 | 0.49 | 3.250E-06 |  | 0.51 | 0.21 | 0.30 | 1.695E-29 |  | 0.44 | 0.19 | 0.25 | 1.333E-26 | |
| cg10959198 | 15 | 93632896 |  | lnc-CHD2-2 | 0.80 | 0.31 | 0.49 | 8.430E-06 |  | 0.68 | 0.24 | 0.44 | 6.086E-36 |  | 0.56 | 0.21 | 0.35 | 1.137E-35 | |
| cg09083579 | 20 | 23030434 |  | lnc-SSTR4-8 | 0.61 | 0.12 | 0.49 | 5.980E-06 |  | 0.41 | 0.11 | 0.30 | 1.298E-17 |  | 0.32 | 0.08 | 0.24 | 1.881E-21 | |
| cg24173246 | 19 | 14897226 |  | lnc-ZNF333-3 | 0.64 | 0.16 | 0.49 | 7.910E-06 |  | 0.54 | 0.11 | 0.43 | 2.388E-25 |  | 0.41 | 0.15 | 0.26 | 1.040E-25 | |
| cg03064067 | 12 | 85306916 |  | lnc-LRRIQ1-1 | 0.88 | 0.40 | 0.48 | 7.690E-06 |  | 0.81 | 0.35 | 0.46 | 1.092E-65 |  | 0.69 | 0.31 | 0.38 | 7.774E-43 | |
| cg25223771 | 8 | 145105503 |  | lnc-SPATC1-1 | 0.67 | 0.19 | 0.48 | 3.980E-06 |  | 0.62 | 0.22 | 0.39 | 1.341E-76 |  | 0.58 | 0.21 | 0.37 | 1.736E-84 | |
| cg14383658 | 10 | 102899262 |  | lnc-LBX1-2 | 0.67 | 0.19 | 0.48 | 7.600E-06 |  | 0.57 | 0.27 | 0.31 | 2.628E-34 |  | 0.50 | 0.20 | 0.31 | 7.192E-48 | |
| cg02442436 | 9 | 112402882 |  | lnc-PALM2-1 | 0.68 | 0.20 | 0.48 | 5.690E-06 |  | 0.48 | 0.23 | 0.25 | 2.483E-19 |  | 0.43 | 0.16 | 0.27 | 3.388E-33 | |
| cg25602490 | 1 | 108507078 |  | VAV3-AS1 | 0.65 | 0.18 | 0.48 | 7.750E-06 |  | 0.47 | 0.09 | 0.38 | 2.307E-23 |  | 0.36 | 0.07 | 0.28 | 2.152E-26 | |
| cg24562819 | 20 | 23029806 |  | lnc-SSTR4-8 | 0.75 | 0.28 | 0.48 | 7.750E-06 |  | 0.62 | 0.21 | 0.41 | 2.280E-49 |  | 0.53 | 0.17 | 0.36 | 2.575E-40 | |
| cg11942956 | 6 | 133562470 |  | lnc-RPS12-6 | 0.65 | 0.18 | 0.47 | 8.860E-06 |  | 0.59 | 0.09 | 0.50 | 1.217E-58 |  | 0.52 | 0.17 | 0.35 | 8.050E-51 | |
| cg09373136 | 17 | 61933544 |  | lnc-PSMC5-7 | 0.25 | 0.72 | -0.47 | 9.020E-06 |  | 0.36 | 0.67 | -0.30 | 2.548E-25 |  | 0.43 | 0.70 | -0.27 | 3.661E-33 | |
| cg10192198 | 9 | 973184 |  | lnc-DMRT3-1 | 0.81 | 0.33 | 0.47 | 8.470E-06 |  | 0.66 | 0.33 | 0.33 | 6.751E-44 |  | 0.55 | 0.27 | 0.28 | 3.641E-34 | |
| cg04594598 | 8 | 145105319 |  | lnc-SPATC1-1 | 0.64 | 0.17 | 0.47 | 7.630E-06 |  | 0.53 | 0.18 | 0.34 | 4.990E-50 |  | 0.47 | 0.14 | 0.33 | 1.332E-59 | |
| cg06664258 | 12 | 103889903 |  | lnc-STAB2-2 | 0.58 | 0.11 | 0.46 | 7.950E-06 |  | 0.47 | 0.20 | 0.27 | 1.055E-15 |  | 0.38 | 0.12 | 0.26 | 7.934E-28 | |
| cg25397597 | 20 | 23030442 |  | lnc-SSTR4-8 | 0.57 | 0.11 | 0.46 | 8.320E-06 |  | 0.37 | 0.04 | 0.34 | 1.706E-16 |  | 0.33 | 0.08 | 0.25 | 3.564E-20 | |
| cg21672843 | 20 | 41818905 |  | lnc-SRSF6-1 | 0.64 | 0.18 | 0.46 | 7.890E-06 |  | 0.54 | 0.12 | 0.43 | 2.332E-42 |  | 0.49 | 0.15 | 0.34 | 4.618E-45 | |
| cg24152605 | 19 | 57050359 |  | lnc-ZNF667-3 | 0.74 | 0.29 | 0.46 | 7.840E-06 |  | 0.43 | 0.07 | 0.36 | 2.248E-14 |  | 0.43 | 0.15 | 0.28 | 8.089E-21 | |
| cg24014202^*^ | 7 | 96636548 |  | DLX6-AS1 | 0.79 | 0.33 | 0.46 | 6.070E-06 |  | 0.60 | 0.34 | 0.26 | 1.173E-40 |  | 0.47 | 0.27 | 0.20 | 8.419E-41 | |
| cg14983606 | 8 | 65493706 |  | lnc-CYP7B1-2 | 0.74 | 0.29 | 0.45 | 5.990E-06 |  | 0.53 | 0.15 | 0.38 | 3.731E-37 |  | 0.44 | 0.13 | 0.31 | 1.059E-32 | |
| cg23916167 | 9 | 37027267 |  | lnc-ZCCHC7-6 | 0.77 | 0.31 | 0.45 | 9.810E-06 |  | 0.64 | 0.36 | 0.28 | 4.692E-39 |  | 0.55 | 0.27 | 0.28 | 1.573E-37 | |
| cg20800509 | 8 | 104513083 |  | lnc-SLC25A32-3 | 0.61 | 0.16 | 0.45 | 6.070E-06 |  | 0.60 | 0.13 | 0.47 | 1.827E-28 |  | 0.52 | 0.18 | 0.34 | 5.625E-30 | |
| cg03700449 | 12 | 103352326 |  | lnc-PAH-3 | 0.63 | 0.18 | 0.45 | 5.450E-06 |  | 0.48 | 0.13 | 0.35 | 4.481E-26 |  | 0.44 | 0.15 | 0.29 | 4.161E-31 | |
| cg01500945 | 16 | 51185672 |  | lnc-HNRNPA1P48-124 | 0.80 | 0.36 | 0.44 | 6.480E-06 |  | 0.68 | 0.26 | 0.43 | 1.980E-52 |  | 0.61 | 0.26 | 0.35 | 1.464E-45 | |
| cg03882242 | 12 | 103889798 |  | lnc-STAB2-2 | 0.82 | 0.38 | 0.44 | 4.210E-06 |  | 0.66 | 0.42 | 0.25 | 1.122E-19 |  | 0.58 | 0.32 | 0.26 | 1.274E-27 | |
| cg03817912^*^ | 20 | 31806172 |  | lnc-CDK5RAP1-1 | 0.21 | 0.64 | -0.43 | 8.900E-06 |  | 0.37 | 0.59 | -0.21 | 2.355E-49 |  | 0.42 | 0.70 | -0.28 | 1.525E-51 | |
| cg23437479 | 3 | 49940353 |  | lnc-RBM6-4 | 0.44 | 0.87 | -0.43 | 8.860E-06 |  | 0.40 | 0.69 | -0.29 | 9.220E-33 |  | 0.48 | 0.81 | -0.33 | 4.087E-37 | |
| cg16201674 | 13 | 95364586 |  | SOX21-AS1 | 0.73 | 0.30 | 0.43 | 7.150E-06 |  | 0.62 | 0.14 | 0.48 | 8.980E-60 |  | 0.56 | 0.22 | 0.34 | 6.795E-45 | |
| cg22277994 | 17 | 71161157 |  | lnc-SSTR2-1 | 0.75 | 0.32 | 0.43 | 2.500E-06 |  | 0.50 | 0.30 | 0.20 | 6.528E-12 |  | 0.42 | 0.21 | 0.21 | 2.064E-20 | |
| cg18335068 | 19 | 53757910 |  | lnc-ZNF677-1 | 0.79 | 0.36 | 0.43 | 7.910E-06 |  | 0.61 | 0.32 | 0.29 | 1.276E-34 |  | 0.53 | 0.26 | 0.27 | 8.161E-33 | |
| cg15397374 | 8 | 49647703 |  | lnc-EFCAB1-5 | 0.79 | 0.37 | 0.42 | 8.190E-06 |  | 0.64 | 0.33 | 0.31 | 8.288E-32 |  | 0.56 | 0.27 | 0.29 | 1.765E-31 | |
| cg14872657 | 7 | 43153216 |  | lnc-PSMA2-1 | 0.55 | 0.13 | 0.42 | 7.010E-06 |  | 0.47 | 0.06 | 0.41 | 6.412E-47 |  | 0.42 | 0.10 | 0.32 | 2.473E-40 | |
| cg26381364 | 12 | 103889772 |  | lnc-STAB2-2 | 0.62 | 0.20 | 0.42 | 9.220E-06 |  | 0.59 | 0.37 | 0.22 | 5.237E-18 |  | 0.54 | 0.31 | 0.23 | 2.013E-28 | |
| cg00419796^*^ | 7 | 137352922 |  | lnc-CREB3L2-2 | 0.22 | 0.64 | -0.42 | 4.720E-06 |  | 0.33 | 0.65 | -0.32 | 2.352E-34 |  | 0.40 | 0.64 | -0.24 | 2.796E-32 | |
| cg23383871 | 20 | 47934987 |  | lnc-DDX27-4 | 0.57 | 0.16 | 0.41 | 6.480E-06 |  | 0.51 | 0.18 | 0.33 | 2.747E-48 |  | 0.47 | 0.16 | 0.31 | 1.706E-53 | |
| cg25649000 | 20 | 41817903 |  | lnc-SRSF6-1 | 0.69 | 0.28 | 0.41 | 3.450E-06 |  | 0.64 | 0.18 | 0.46 | 2.434E-55 |  | 0.54 | 0.21 | 0.33 | 1.399E-41 | |
| cg13702005 | 12 | 103889789 |  | lnc-STAB2-2 | 0.72 | 0.32 | 0.40 | 7.050E-06 |  | 0.64 | 0.39 | 0.25 | 6.885E-16 |  | 0.56 | 0.31 | 0.25 | 4.305E-27 | |
| cg08258526 | 8 | 49647734 |  | lnc-EFCAB1-5 | 0.82 | 0.42 | 0.40 | 5.680E-06 |  | 0.65 | 0.36 | 0.29 | 2.720E-31 |  | 0.58 | 0.30 | 0.28 | 5.550E-33 | |
| cg08464824 | 19 | 56904977 |  | ZNF582-AS1 | 0.55 | 0.16 | 0.39 | 9.530E-06 |  | 0.42 | 0.16 | 0.26 | 1.904E-22 |  | 0.39 | 0.11 | 0.28 | 8.626E-38 | |
| cg24190603 | 6 | 84418433 |  | lnc-RIPPLY2-8 | 0.86 | 0.47 | 0.39 | 9.310E-06 |  | 0.75 | 0.28 | 0.46 | 8.013E-94 |  | 0.65 | 0.26 | 0.39 | 9.774E-50 | |
| cg20248516 | 2 | 119602901 |  | lnc-C1QL2-3 | 0.78 | 0.40 | 0.39 | 3.250E-06 |  | 0.68 | 0.33 | 0.35 | 2.108E-55 |  | 0.56 | 0.30 | 0.26 | 5.862E-38 | |
| cg20285745 | 12 | 117798627 |  | lnc-KSR2-2 | 0.77 | 0.39 | 0.38 | 4.890E-06 |  | 0.59 | 0.24 | 0.35 | 4.483E-42 |  | 0.46 | 0.21 | 0.24 | 1.158E-30 | |
| cg01515802 | 19 | 54876749 |  | lnc-LILRA4-2 | 0.22 | 0.61 | -0.38 | 8.750E-06 |  | 0.15 | 0.38 | -0.23 | 2.291E-30 |  | 0.23 | 0.50 | -0.26 | 1.931E-32 | |
| cg05031521 | 16 | 1030561 |  | lnc-LMF1-3 | 0.60 | 0.22 | 0.38 | 8.760E-06 |  | 0.45 | 0.11 | 0.34 | 1.643E-31 |  | 0.41 | 0.16 | 0.25 | 4.833E-31 | |
| cg01112805 | 13 | 39262298 |  | LINC00437 | 0.82 | 0.44 | 0.38 | 7.330E-06 |  | 0.64 | 0.33 | 0.31 | 7.185E-47 |  | 0.57 | 0.31 | 0.26 | 4.438E-42 | |
| cg26478990^*^ | 16 | 54967686 |  | lnc-IRX5-1 | 0.88 | 0.51 | 0.37 | 2.190E-06 |  | 0.53 | 0.21 | 0.33 | 4.778E-36 |  | 0.45 | 0.22 | 0.23 | 1.839E-38 | |
| cg05379302 | 1 | 11540200 |  | lnc-DISP3-1 | 0.61 | 0.24 | 0.37 | 9.520E-06 |  | 0.53 | 0.25 | 0.28 | 2.288E-22 |  | 0.42 | 0.18 | 0.24 | 1.316E-26 | |
| cg26709300 | 16 | 30106682 |  | lnc-TBX6-1 | 0.82 | 0.45 | 0.37 | 7.150E-06 |  | 0.75 | 0.40 | 0.34 | 6.249E-84 |  | 0.69 | 0.38 | 0.30 | 5.656E-55 | |
| cg23572908 | 7 | 158937969 |  | lnc-VIPR2-1 | 0.86 | 0.51 | 0.35 | 6.570E-06 |  | 0.76 | 0.29 | 0.48 | 1.337E-72 |  | 0.68 | 0.28 | 0.39 | 5.202E-43 | |
| cg20028827 | 13 | 79169823 |  | lnc-SLAIN1-11 | 0.81 | 0.47 | 0.34 | 9.990E-06 |  | 0.71 | 0.44 | 0.27 | 7.017E-33 |  | 0.63 | 0.41 | 0.22 | 5.827E-33 | |
| cg03957884 | 13 | 23471850 |  | LINC00621 | 0.46 | 0.80 | -0.34 | 9.540E-06 |  | 0.54 | 0.89 | -0.35 | 2.255E-42 |  | 0.58 | 0.86 | -0.28 | 6.655E-46 | |
| cg21516937^*^ | 6 | 127210160 |  | lnc-ECHDC1-1 | 0.13 | 0.43 | -0.30 | 8.750E-06 |  | 0.39 | 0.15 | 0.24 | 2.263E-30 |  | 0.33 | 0.13 | 0.20 | 2.098E-34 | |
| ^*^The new probes in 850K array was cross-validated by the average beta value of the promoter regions of the target genes in 450K array. | | | | | | | | | | | | | | | | | | | |
| CHR, chromosome; NAT, normal adjacent tissue; *P*_adj_, adjusted *P* value with Benjamini-Hochberg method. | | | | | | | | | | | | | | | | | | | |

| **Table S5.** Blood-based *DLX6-AS1* methylation differences between colorectal cancer patients, adenoma patients and healthy controls | | | | | | | | | | | | | | | |
| --- | --- | --- | --- | --- | --- | --- | --- | --- | --- | --- | --- | --- | --- | --- | --- |
| CpG unit | CRC/AD/CON, n |  | Methylation level | | | *P ^a^* |  | CRC vs. AD | |  | CRC vs. CON | |  | AD vs. CON | |
|  |  |  | CRC | AD | CON |  |  | Δß | *P ^b^* |  | Δß | *P ^b^* |  | Δß | *P ^b^* |
| Mean | 60/60/60 |  | 0.15 | 0.16 | 0.15 | 0.496 |  | -0.01 | - |  | -0.01 | - |  | 0.00 | - |
| CpG_1 | 57/56/60 |  | 0.40 | 0.39 | 0.41 | 0.865 |  | 0.01 | - |  | -0.01 | - |  | -0.02 | - |
| CpG_2.3 | 60/60/60 |  | 0.20 | 0.20 | 0.17 | **0.017** |  | 0.00 | 1.000 |  | 0.03 | **0.038** |  | 0.03 | **0.041** |
| CpG_4 | 60/60/60 |  | 0.08 | 0.11 | 0.10 | **0.002** |  | -0.03 | **0.002** |  | -0.02 | 0.063 |  | 0.01 | 0.694 |
| CpG_5.6.7 | 59/60/60 |  | 0.12 | 0.12 | 0.11 | 0.338 |  | 0.00 | - |  | 0.01 | - |  | 0.01 | - |
| CpG_11 | 60/60/60 |  | 0.07 | 0.10 | 0.08 | 0.055 |  | -0.02 | - |  | 0.00 | - |  | 0.02 | - |
| CpG_12.13.14 | 59/60/60 |  | 0.11 | 0.13 | 0.11 | 0.114 |  | -0.02 | - |  | 0.00 | - |  | 0.01 | - |
| CpG_15 | 60/60/60 |  | 0.12 | 0.15 | 0.13 | 0.117 |  | -0.02 | - |  | -0.01 | - |  | 0.02 | - |
| CpG_17 | 60/60/60 |  | 0.34 | 0.33 | 0.41 | 0.114 |  | 0.01 | - |  | -0.07 | - |  | -0.08 | - |
| CpG_18 | 59/59/60 |  | 0.09 | 0.10 | 0.09 | 0.305 |  | -0.01 | - |  | 0.00 | - |  | 0.01 | - |
| CpG_20 | 60/60/60 |  | 0.08 | 0.11 | 0.10 | **0.002** |  | -0.03 | **0.002** |  | -0.02 | 0.063 |  | 0.01 | 0.694 |
| CpG_21 | 56/59/59 |  | 0.26 | 0.21 | 0.21 | **0.005** |  | 0.06 | **0.013** |  | 0.05 | **0.017** |  | 0.00 | 1.000 |
| CpG_22 | 60/60/60 |  | 0.05 | 0.06 | 0.05 | 0.143 |  | -0.02 | - |  | -0.01 | - |  | 0.01 | - |
| CpG_23 | 60/59/60 |  | 0.02 | 0.05 | 0.03 | **0.037** |  | -0.03 | **0.040** |  | -0.01 | 1.000 |  | 0.02 | 0.201 |
| AD, adenoma; CON, healthy controls; CRC, colorectal cancer. | | | | | | | | | | | | | | | |
| ^a^ ANOVA. | | | | | | | | | | | | | | | |
| ^b^ Bonferroni *t* test.  Bold values: Statistically significant. | | | | | | | | | | | | | | | |

| **Table S6.** Paired comparisons of *DLX6-AS1* methylation levels between peripheral blood and local tissue from the same patient | | | | | | | | | | | | | | | |
| --- | --- | --- | --- | --- | --- | --- | --- | --- | --- | --- | --- | --- | --- | --- | --- |
| CpG unit | Colorectal cancer | | | | | | |  | Adenoma | | | | | | |
|  | Methylation level | | | |  | *r* | *P ^a^* |  | Methylation level | | | |  | *r* | *P ^a^* |
|  | n | Tissue | Blood | Δß |  |  |  |  | n | Tissue | Blood | Δß |  |  |  |
| Mean | 60 | 0.40 | 0.15 | 0.26 |  | -0.12 | 0.362 |  | 60 | 0.39 | 0.16 | 0.23 |  | 0.02 | 0.893 |
| CpG_1 | 56 | 0.27 | 0.40 | -0.12 |  | 0.05 | 0.740 |  | 52 | 0.33 | 0.40 | -0.07 |  | 0.26 | 0.065 |
| CpG_2.3 | 60 | 0.43 | 0.20 | 0.24 |  | -0.11 | 0.389 |  | 60 | 0.47 | 0.20 | 0.27 |  | -0.01 | 0.958 |
| CpG_4 | 60 | 0.42 | 0.08 | 0.34 |  | -0.15 | 0.246 |  | 60 | 0.40 | 0.11 | 0.28 |  | -0.01 | 0.919 |
| CpG_5.6.7 | 59 | 0.38 | 0.12 | 0.27 |  | 0.10 | 0.449 |  | 59 | 0.36 | 0.12 | 0.25 |  | -0.18 | 0.179 |
| CpG_11 | 60 | 0.44 | 0.07 | 0.37 |  | 0.03 | 0.825 |  | 60 | 0.40 | 0.10 | 0.30 |  | -0.10 | 0.442 |
| CpG_12.13.14 | 59 | 0.41 | 0.11 | 0.30 |  | 0.14 | 0.280 |  | 60 | 0.37 | 0.13 | 0.24 |  | -0.10 | 0.449 |
| CpG_15 | 60 | 0.44 | 0.12 | 0.32 |  | 0.04 | 0.741 |  | 60 | 0.40 | 0.15 | 0.25 |  | -0.07 | 0.583 |
| CpG_17 | 60 | 0.47 | 0.34 | 0.12 |  | -0.06 | 0.635 |  | 60 | 0.43 | 0.33 | 0.09 |  | 0.04 | 0.769 |
| CpG_18 | 59 | 0.41 | 0.09 | 0.32 |  | 0.19 | 0.157 |  | 59 | 0.41 | 0.10 | 0.32 |  | -0.01 | 0.968 |
| CpG_20 | 60 | 0.42 | 0.08 | 0.34 |  | -0.15 | 0.246 |  | 60 | 0.40 | 0.11 | 0.28 |  | -0.01 | 0.919 |
| CpG_21 | 55 | 0.49 | 0.27 | 0.22 |  | -0.11 | 0.430 |  | 58 | 0.46 | 0.21 | 0.25 |  | -0.18 | 0.166 |
| CpG_22 | 60 | 0.37 | 0.05 | 0.32 |  | -0.03 | 0.831 |  | 60 | 0.38 | 0.06 | 0.31 |  | -0.08 | 0.525 |
| CpG_23 | 60 | 0.31 | 0.02 | 0.29 |  | -0.13 | 0.338 |  | 59 | 0.26 | 0.05 | 0.22 |  | -0.16 | 0.234 |
| ^a^ Pearson correlation analyses. | | | | | | | | | | | | | | | |

**3. Supplementary Figures**


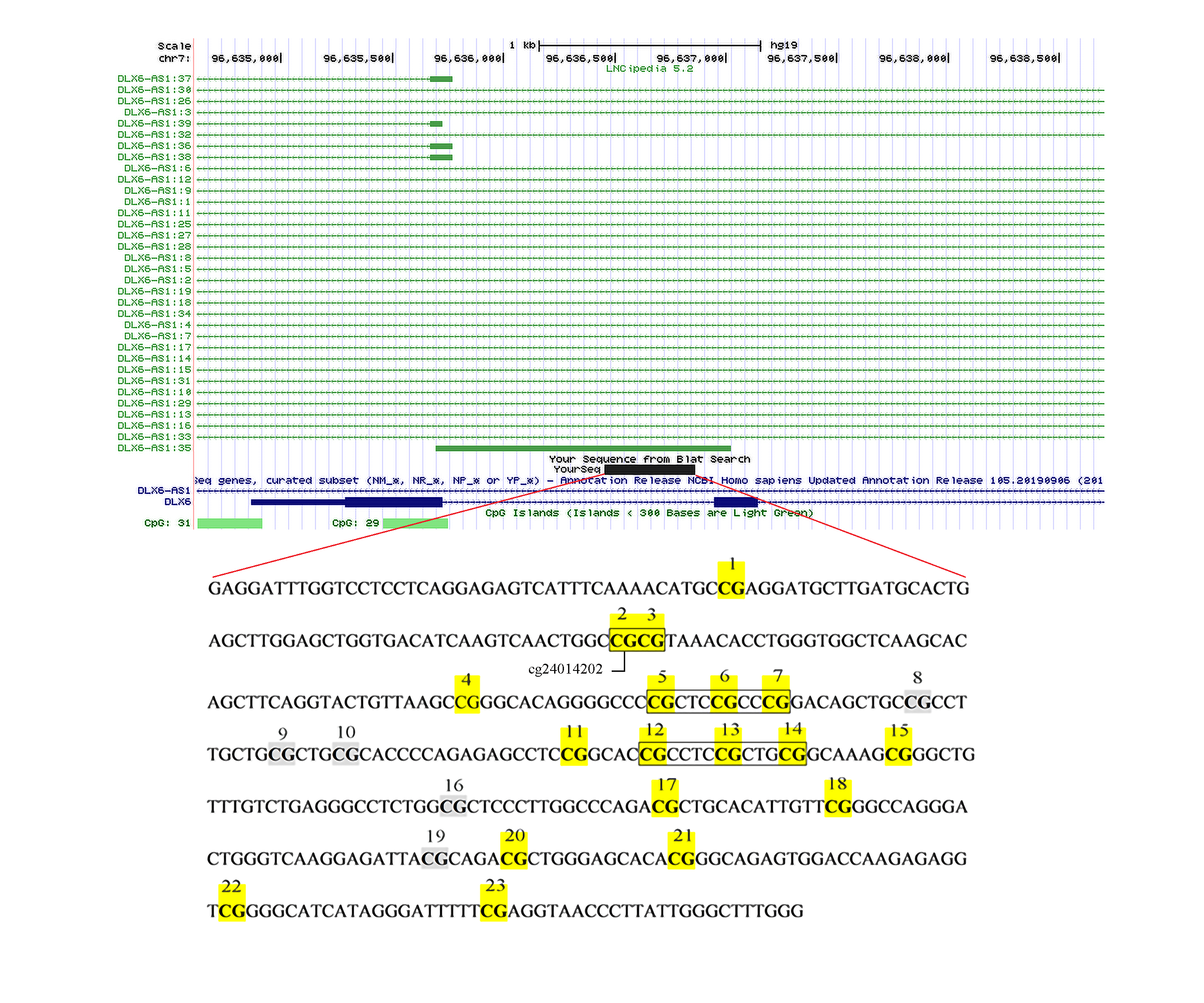


**Figure S1.** **Amplified fragment of *DLX6-AS1* by MassARRAY EpiTYPER.** The position of the studied region (chr7:96636455-96636863, GRCh37/hg19 Assembly) is depicted as a black box. The measured CpG sites in the amplified fragment are shown in bold, among which CpG_2 corresponds to cg24014202 in the 850K array. Thirteen CpG units (18 CpG sites) highlighted in yellow were successfully measured, and the other 5 CpG sites marked in gray could not be quantified reliably and were filtered before the subsequent analysis.


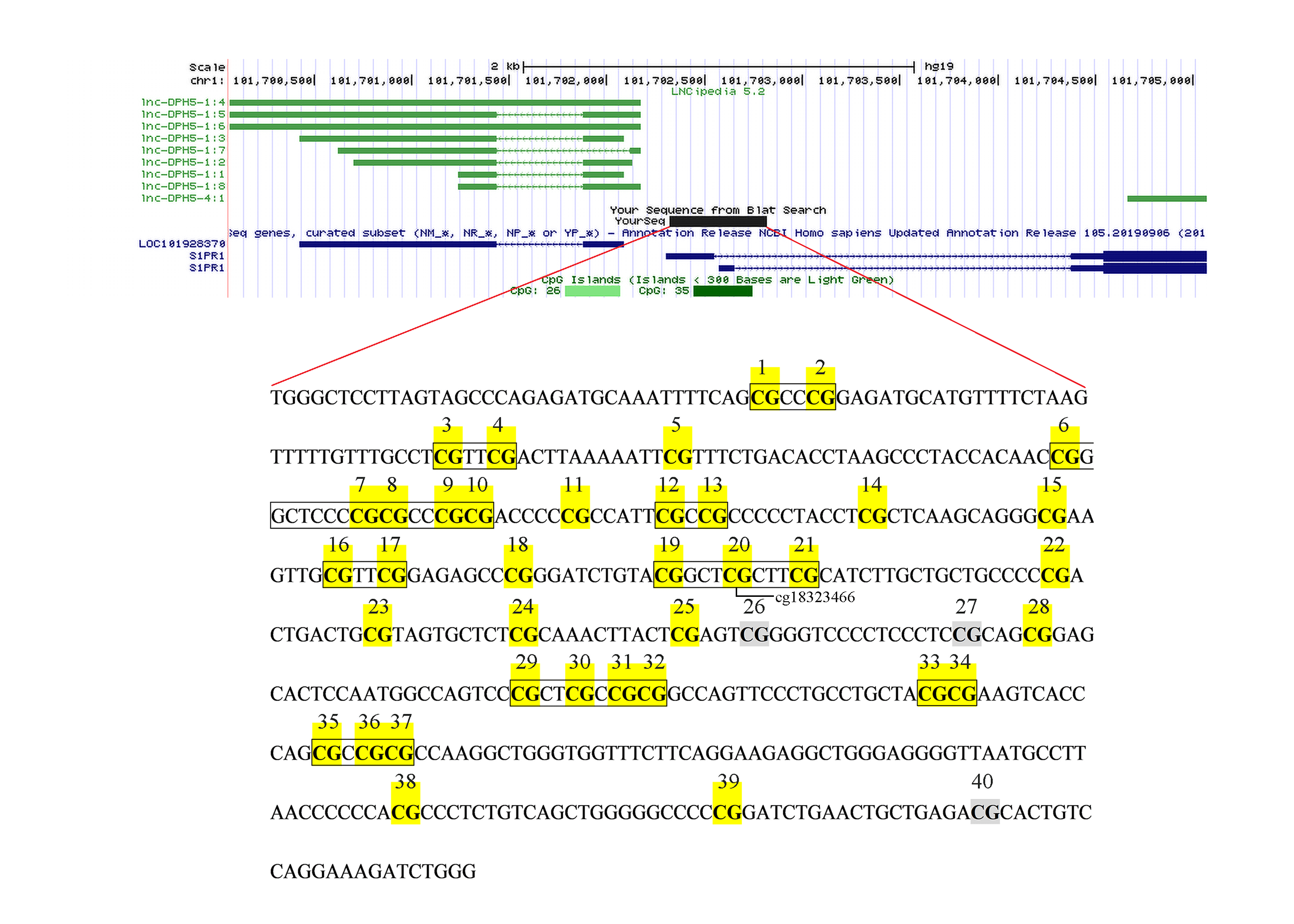


**Figure S2.** **Amplified fragment of *lnc-DPH5-1* by MassARRAY EpiTYPER.** The position of the studied region (chr1:101702320-101702819, GRCh37/hg19 Assembly) is depicted as a black box. The measured CpG sites in the amplified fragment are shown in bold, among which CpG_20 correspond to cg18323466 in the 850K array. Twenty one CpG units (37 CpG sites) highlighted in yellow were successfully measured, and the other 3 CpG sites marked in gray could not be quantified reliably and were filtered before the subsequent analysis.

**
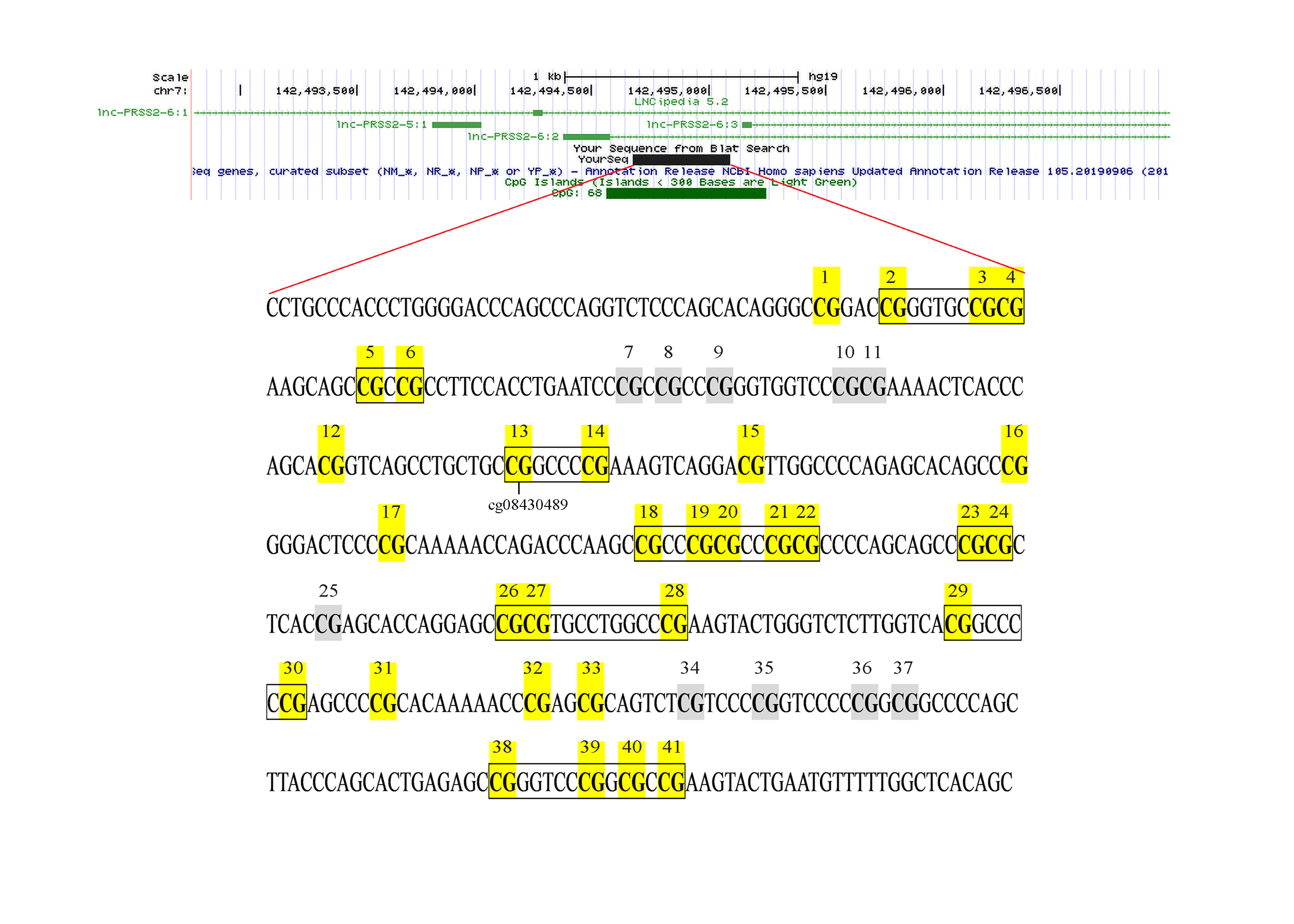
**

**Figure S3.** **Amplified fragment of *lnc-PRSS2-6* by MassARRAY EpiTYPER.** The position of the studied region (chr7:142494676-142495093, GRCh37/hg19 Assembly) is depicted as a black box. The measured CpG sites in the amplified fragment are shown in bold, among which CpG_13 correspond to cg08430489 in the 850K array. Sixteen CpG units (31 CpG sites) highlighted in yellow were successfully measured, and the other 10 CpG sites marked in gray could not be quantified reliably and were filtered before the subsequent analysis.

**
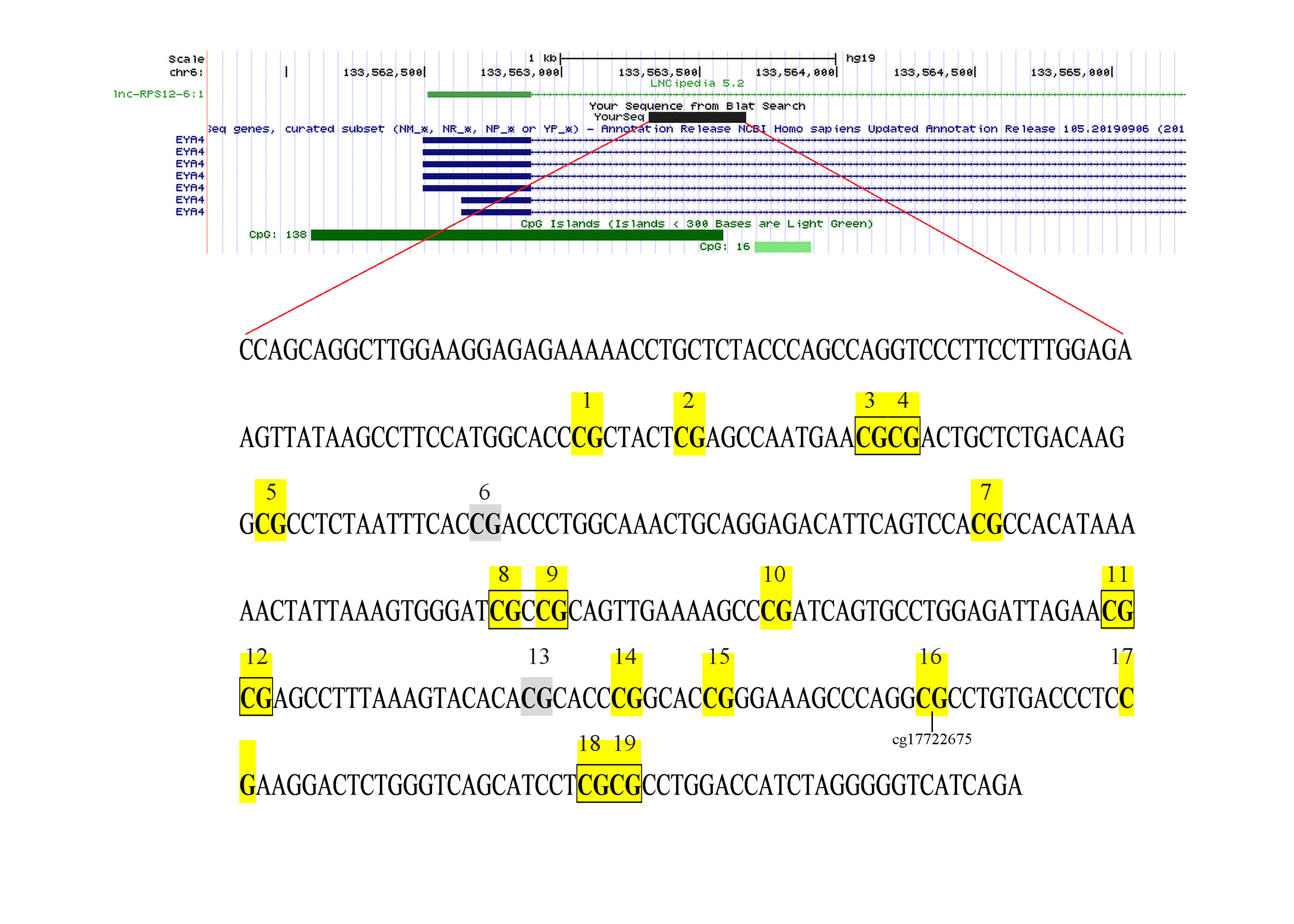
**

**Figure S4.** **Amplified fragment of *lnc-RPS12-6* by MassARRAY EpiTYPER.** The position of the studied region (chr6:133563316-133563670, GRCh37/hg19 Assembly) is depicted as a black box. The measured CpG sites in the amplified fragment are shown in bold, among which CpG_16 correspond to cg17722675 in the 850K array. Thirteen CpG units (17 CpG sites) highlighted in yellow were successfully measured, and the other 2 CpG sites marked in gray could not be quantified reliably and were filtered before the subsequent analysis.


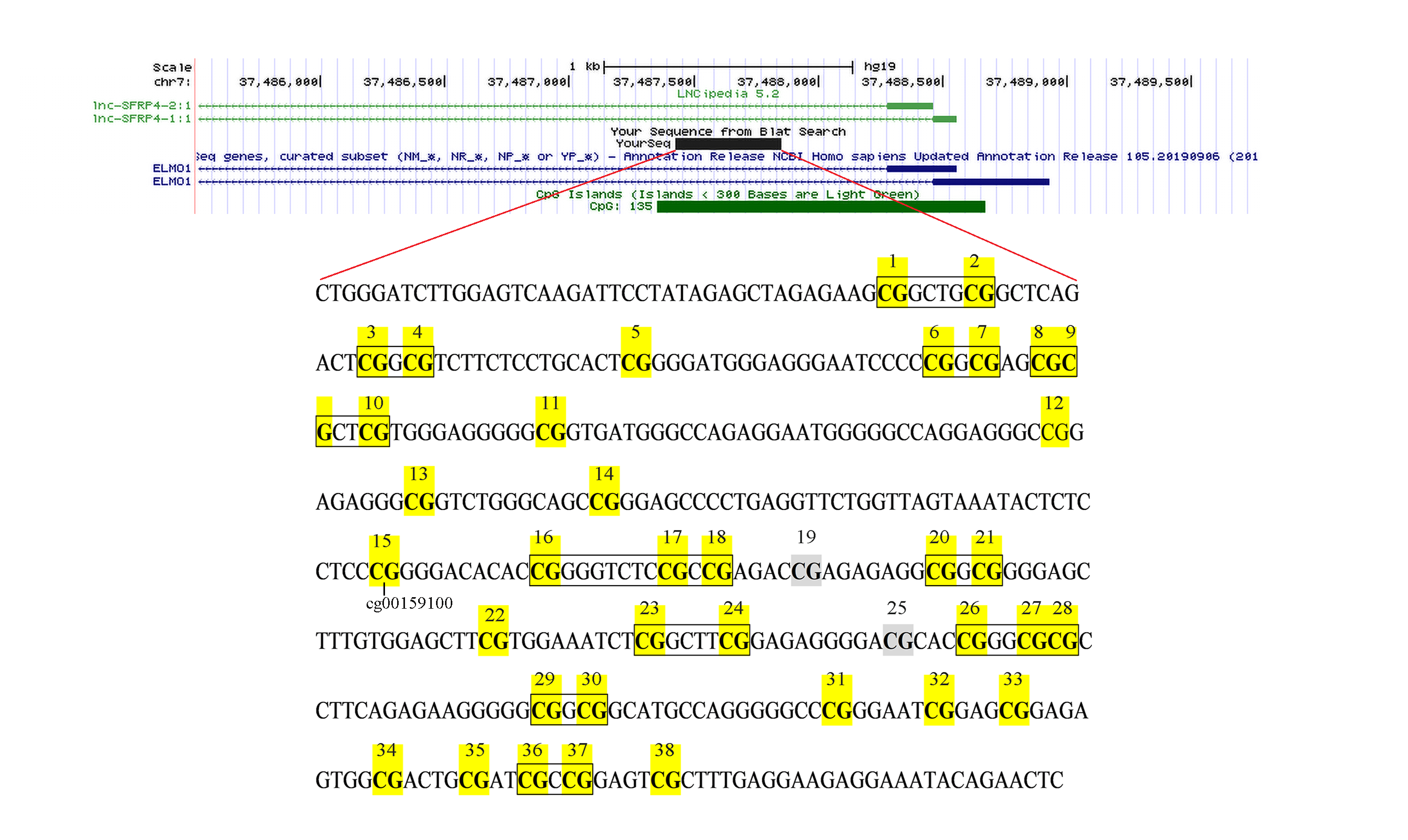


**Figure S5.** **Amplified fragment of *lnc-SFRP4-2* by MassARRAY EpiTYPER.** The position of the studied region (chr7:37487427-37487853, GRCh37/hg19 Assembly) is depicted as a black box. The measured CpG sites in the amplified fragment are shown in bold, among which CpG_15 correspond to cg00159100 in the 850K array. Twenty three CpG units (36 CpG sites) highlighted in yellow were successfully measured, and the other 2 CpG sites marked in gray could not be quantified reliably and were filtered before the subsequent analysis.

**
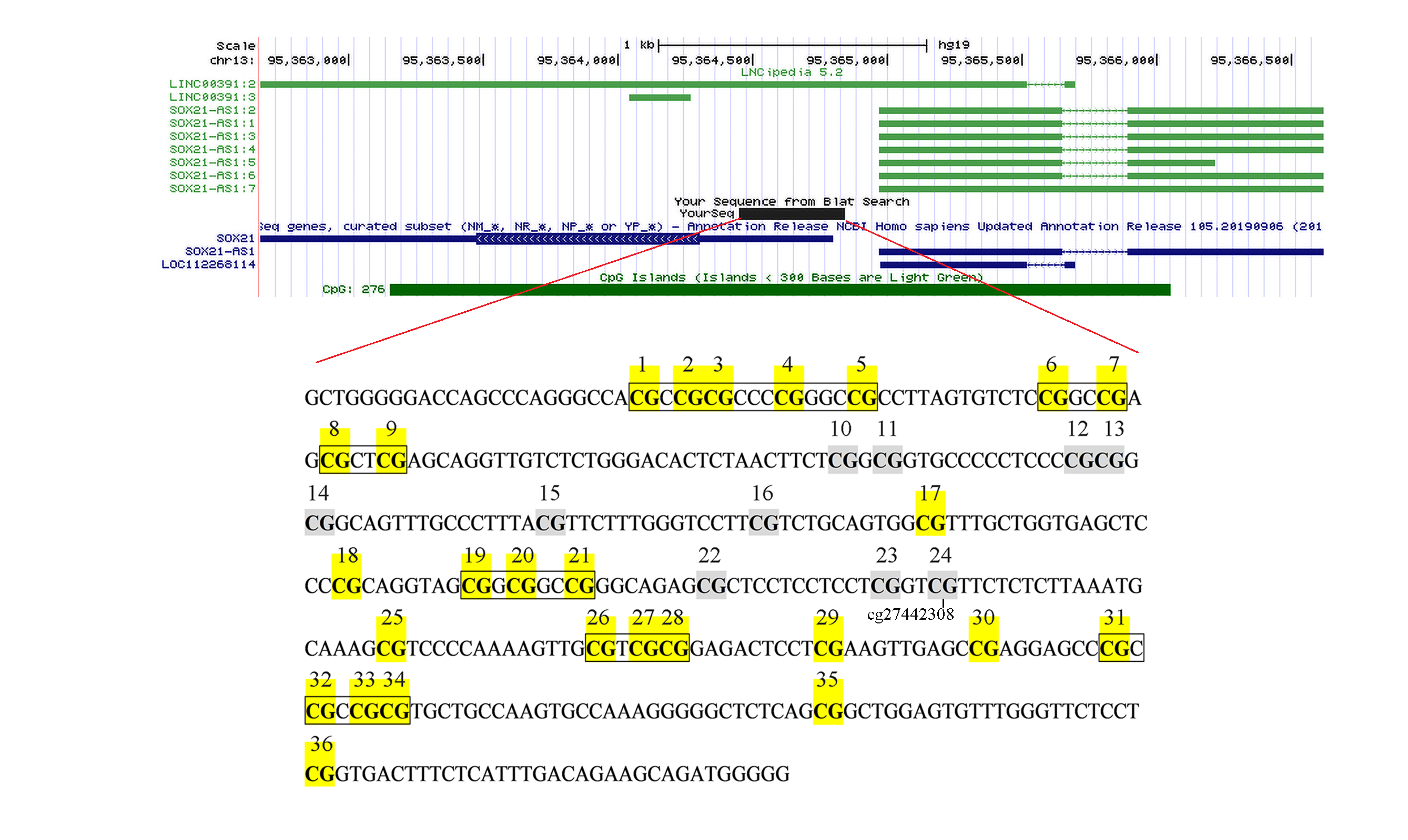
**

**Figure S6.** **Amplified fragment of *SOX21-AS1* by MassARRAY EpiTYPER.** The position of the studied region (chr13:95364450-95364844, GRCh37/hg19 Assembly) is depicted as a black box. The measured CpG sites in the amplified fragment are shown in bold, among which CpG_24 correspond to cg27442308 in the 850K array. Thirteen CpG units (26 CpG sites) highlighted in yellow were successfully measured, and the other 10 CpG sites marked in gray could not be quantified reliably and were filtered before the subsequent analysis.**
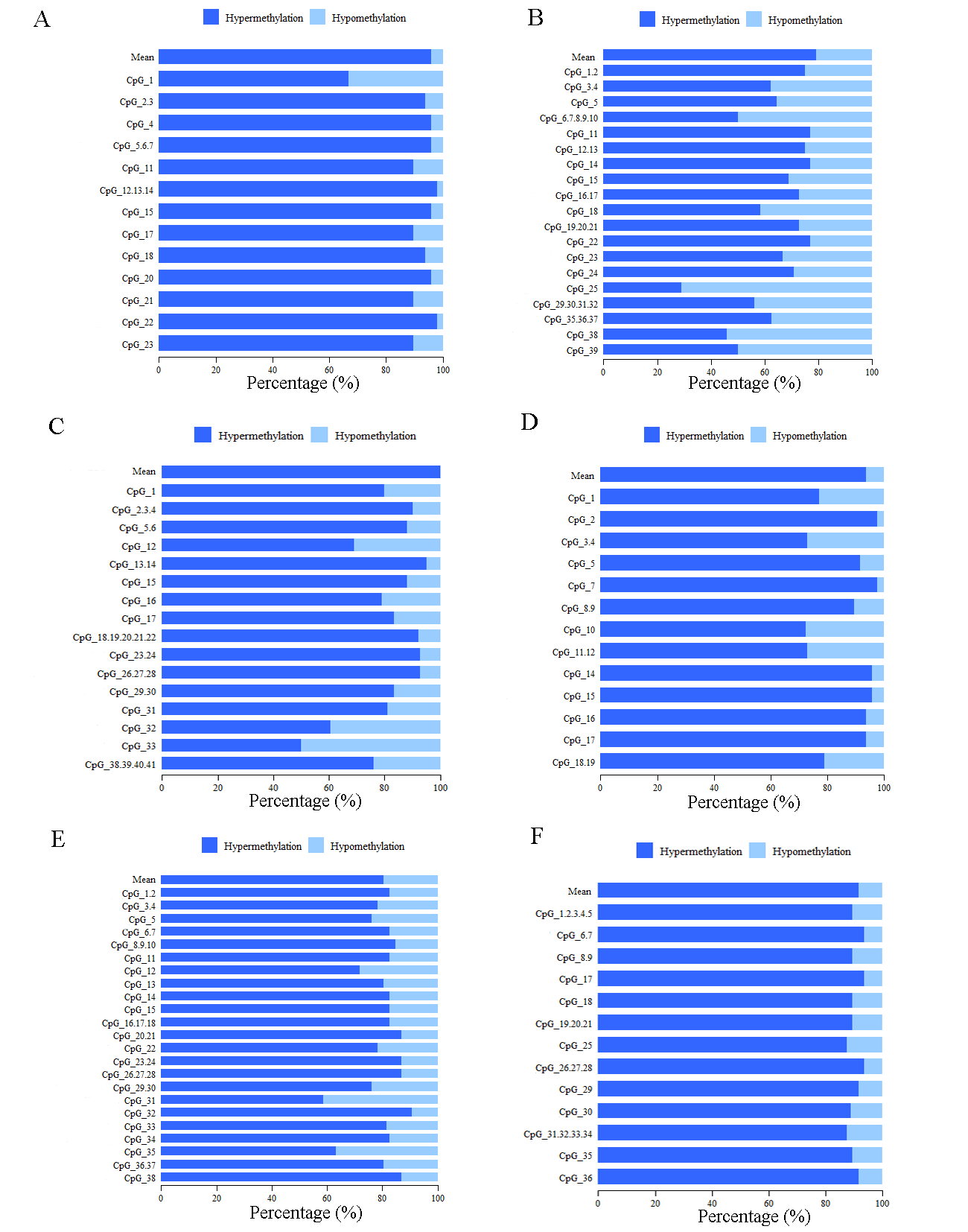
**

**Figure S7**. **Percentages of patients in cohort II with a hyper or hypomethylation status in lesion tissues compared to adjacent normal tissues. A.** *DLX6-AS1*. **B.** *lnc-DPH5-1*. **C.** *lnc-PRSS2-6*. **D.** *lnc-RPS12-6*. **E.** *lnc-SFRP4-2*. **F.** *SOX21-AS1*.


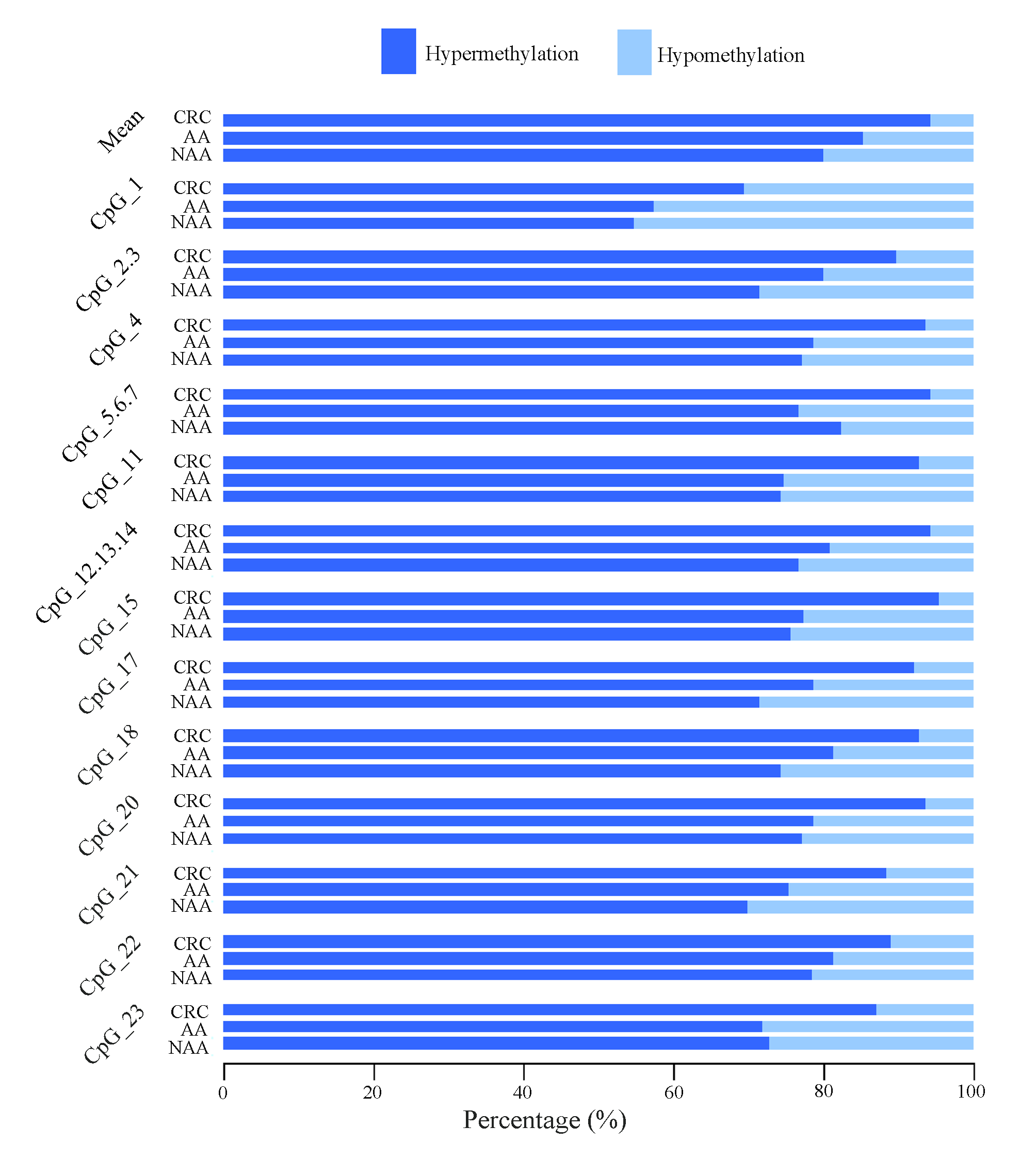


**Figure S8**. **Percentages of patients in cohort III with a *DLX6-AS1* hyper or hypomethylation status in lesion tissues compared to adjacent normal tissues.** AA, advanced adenoma; CRC, colorectal cancer; NAA, nonadvanced adenoma.


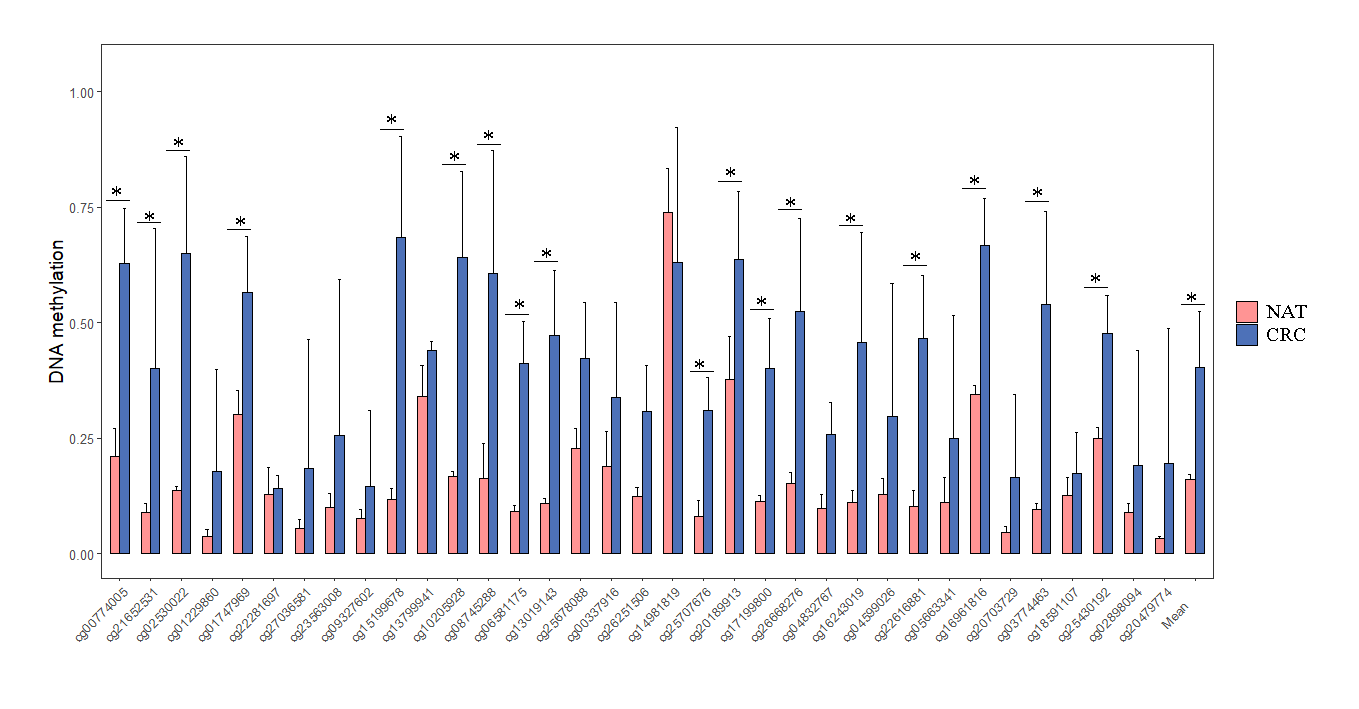


**Figure S9**. **Methylation status of 35 CpG sites of *DLX6-AS1* promoter in cell-free DNA samples from 3 colorectal cancer patients and 4 healthy controls in the GEO dataset.** CRC, colorectal cancer; NAT, histologically normal tissue adjacent to the lesion.


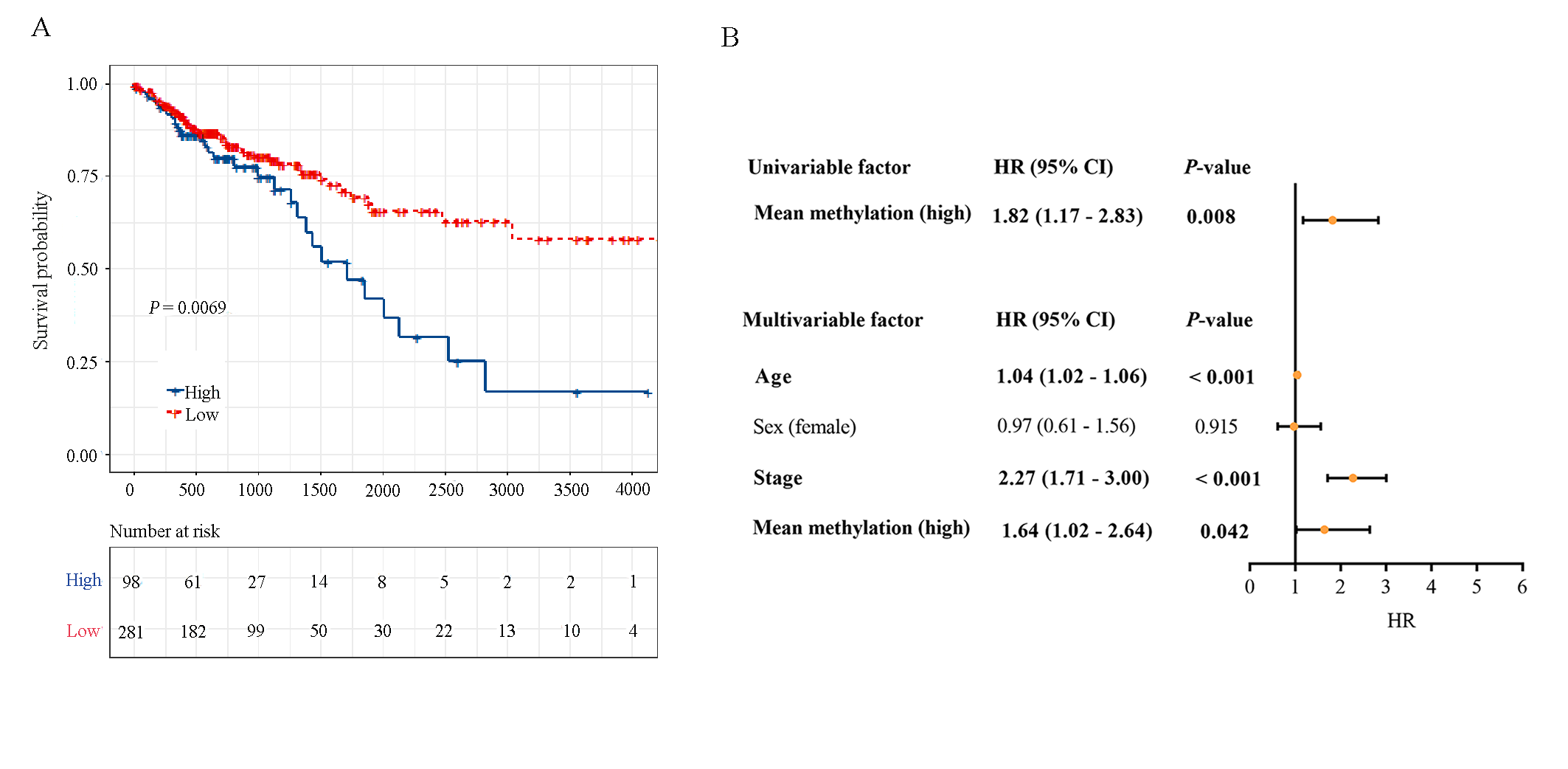


**Figure S10**. **Prognosis prediction of *DLX6-AS1* promoter methylation on CRC overall survival in TCGA dataset. A.** Kaplan-Meier estimation of the overall survival of CRC patients (N = 379) using the mean methylation levels of *DLX6-AS1*. The blue line indicates the group with a high methylation level. The red line indicates the group with a low methylation level. **B.** Univariate and multivariate Cox regression analysis with the methylation level. Multivariate Cox regression analysis were adjusted for age, sex and TNM stage. Orange solid dots represent the point estimation of the hazard ratio (HR) of disease-specific death, and the open-ended horizontal lines represent the 95% confidence intervals (CIs).

**
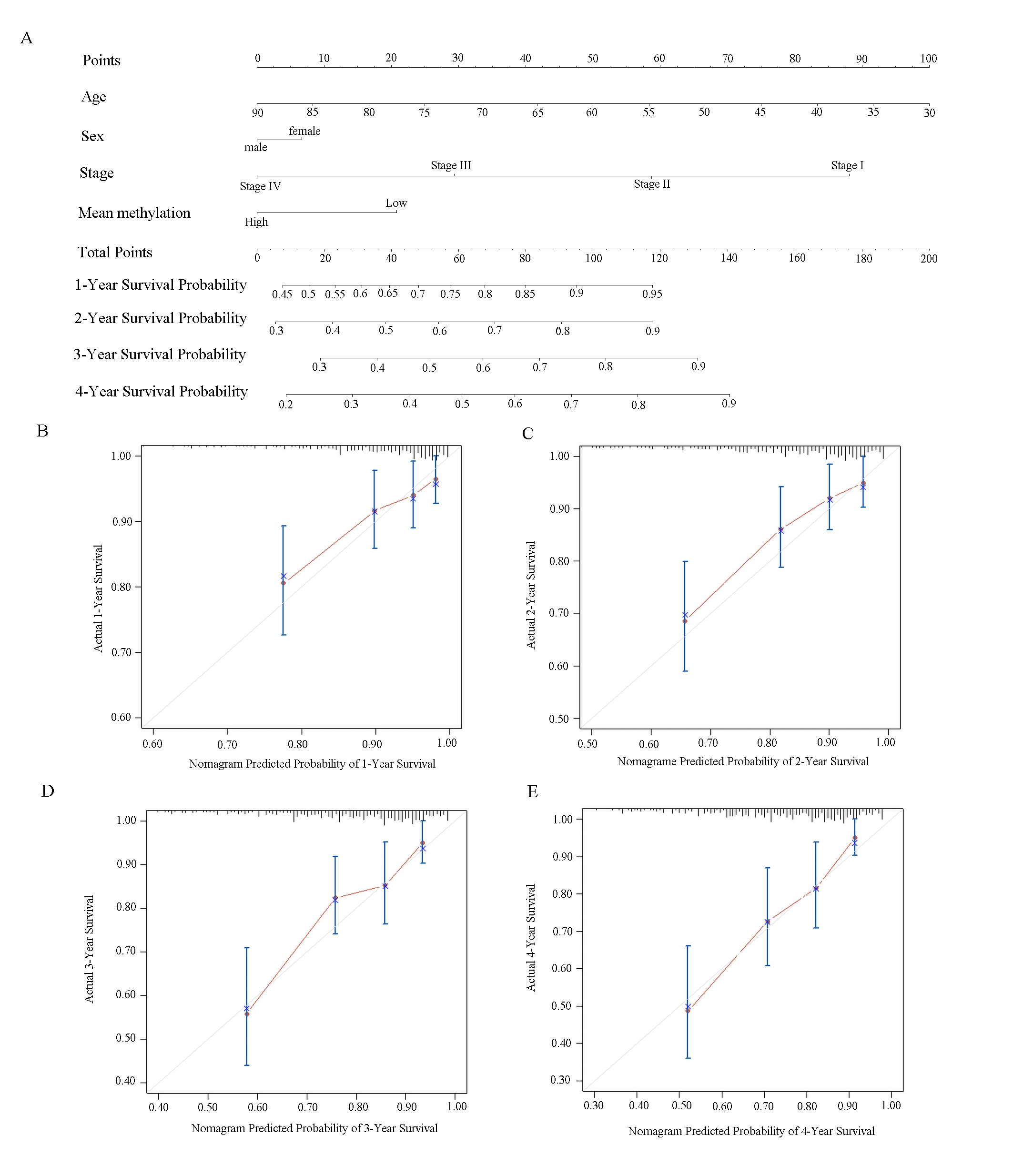
**

**Figure S11**. **Establishment of a nomogram for survival prediction in TCGA dataset. A.** Nomogram to predict the 1-, 2-, 3-, and 4-year overall survival of CRC patients. Calibration curves of **B.**1-year, **C.**2-year, **D.**3-year, and **E.**4-year survival nomogram model. The gray line represents the ideal predictive model, and the red line represents the observed model.
